# Viral Infection at High Magnification: 3D Electron Microscopy Methods to Analyze the Architecture of Infected Cells

**DOI:** 10.3390/v7122940

**Published:** 2015-12-03

**Authors:** Inés Romero-Brey, Ralf Bartenschlager

**Affiliations:** 1 Department of Infectious Diseases, Molecular Virology, Heidelberg University, Im Neuenheimer Feld 345, 69120 Heidelberg, Germany; 2 German Center for Infection Research, Heidelberg University, 69120, Heidelberg, Germany

**Keywords:** transmission electron microscopy, electron tomography, serial sectioning, scanning transmission electron microscopy, serial block face-scanning electron microscopy, focus ion beam-scanning electron microscopy, virus-host interactions, cell membranes, ultrastructure, cryo-EM

## Abstract

As obligate intracellular parasites, viruses need to hijack their cellular hosts and reprogram their machineries in order to replicate their genomes and produce new virions. For the direct visualization of the different steps of a viral life cycle (attachment, entry, replication, assembly and egress) electron microscopy (EM) methods are extremely helpful. While conventional EM has given important information about virus-host cell interactions, the development of three-dimensional EM (3D-EM) approaches provides unprecedented insights into how viruses remodel the intracellular architecture of the host cell. During the last years several 3D-EM methods have been developed. Here we will provide a description of the main approaches and examples of innovative applications.

## 1. Virology and Electron Microscopy (EM)

Studies in virology go hand in hand with the development of microscopy techniques. Among them, electron microscopy (EM) has played a major role due to the small size of virus particles that, with very few exceptions, cannot be visualized by conventional light microscopy [1,2,3,4]. Prior to the invention of the electron microscope in 1931 by the German engineers Ernst Ruska and Max Knoll [5], viruses were detected indirectly e.g., by means of the cytopathic effect they cause in infected cells or through clinical manifestations. However, the availability of EM enabled us to visualize and identify many infectious agents causing diseases or living “in symbiosis” with other organisms. Thus, during the 20^th^ century EM has been a standard technique for virus diagnosis (reviews by [6,7,8,9]). Since they are simpler and faster, molecular biology techniques like PCR or ELISA with higher throughput are more commonly used. Nevertheless, EM remains essential to identify e.g., unknown emerging viruses, for which no primers, antibodies or probes are available [10]. This is due to the fact that EM is a generic approach and has the potential to detect all viral particles (“catch-all”) present in a sample [11,12].

Currently, a main application of EM in virology is visualization of the different steps of the viral life cycle (attachment, entry, replication, assembly and egress) and the study of the ultrastructure of the infected cell (recently reviewed in [13]). In fact, understanding virus-induced modifications of a targeted subcellular organelle is not only critical to elucidate the function of different viral proteins, but also to design novel antiviral drugs and eventually vaccines. Furthermore, with the implementation of novel EM techniques, we are now able to unravel the three-dimensional (3D) architecture of cellular organelles and even complete virus-infected cells. In this review we have compiled information about the different EM approaches with a focus on methods allowing the visualization of cellular structures in 3D.

## 2. Preparation of Cells for EM

Preparation of cells for EM should follow one major goal, *i.e.*, to preserve the ultrastructure in a state that is as close as possible to a snapshot of the living state. Quoting Gareth Griffiths, a pioneer in EM [14]: “the cell structure should be preserved exactly as it was in the living state and should be visualized at the resolution limit of the electron microscope”. To achieve this goal, several methods are available and standard recipes have been established. Although they are of great help for routine applications, they must be frequently modified when addressing particular biological questions. Thus, it is advisable to first get in contact with an EM specialist to choose the method of choice and to prepare the sample accordingly. For readers interested in a broader overview of different EM methods we recommend the reading of earlier published books by Hayak [15], Steinbrecht and Zierold [16], Griffiths [14], and Verkleij and Leunissen [17]. Furthermore, for a quick introduction to EM techniques applied to the study of viruses we also recommend the chapter written by Michael Laue [18].

**Figure 1:**
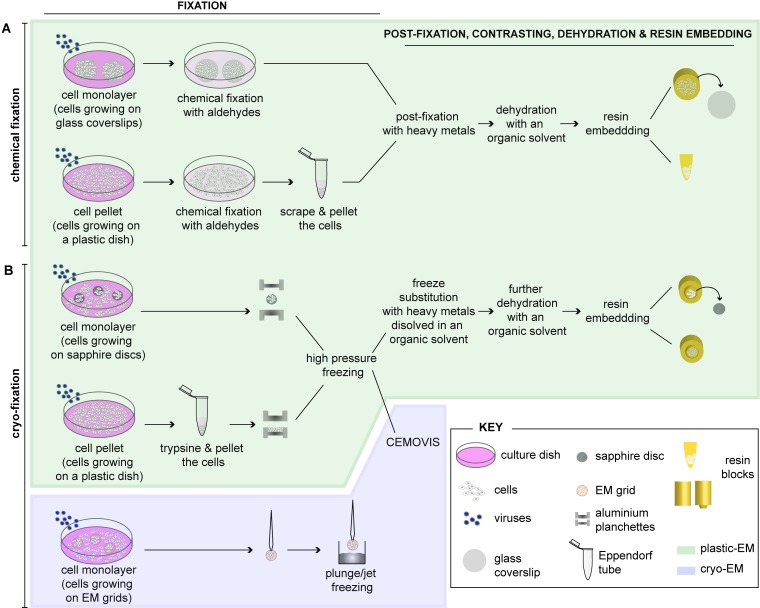
Schematic representation of the different methods for preparing virus-infected cells for electron microscopy (EM). Adherent cells can be either chemically fixed with aldehydes (**A**) or fast frozen by high-pressure freezing (HPF) or plunge/jet freezing (**B**). (**A**) After chemical fixation, infected cells can be further processed as a cell monolayer (grown on glass coverslips) or be pelleted prior to further processing for EM. Post-fixation is done with heavy metals (e.g., osmium tetroxide (OsO_4_) and uranyl acetate (UA)), samples are dehydrated with increasing concentrations of an organic solvent (e.g., ethanol or acetone) and embedded into a plastic resin (plastic-EM; highlighted in green). Coverslips must be removed from the polymerized resin block by successive immersions in liquid nitrogen and hot water; (**B**) For rapid immobilization of cells by HPF, they must be grown as monolayers on sapphire (or aclar, not shown) discs, which are clamped in-between two aluminium planchettes and then loaded into a HPF machine for rapid freezing. Alternatively, cell pellets are directly placed into the aluminium planchettes or into capillary tubes (not shown) for freezing. Frozen cells can be subjected to freeze substitution (FS), dehydration with an organic solvent and resin embedding (plastic-EM; highlighted in green). Alternatively, high-pressure frozen cell pellets can be further analyzed by CEMOVIS (cryo-electron microscopy of vitreous sections) (cryo-EM, highlighted in violet). Cells growing on EM grids can be also plunge/jet frozen and analyzed directly by cryo-EM (highlighted in violet). Both cryo-EM approaches, which do not require further processing of the cells, allow visualizing cells in their closest-to-native status. Further details about these approaches can be found in the main text.

The standard method to prepare cells for routine EM involves the following steps: fixation, embedding and sectioning. These will be extensively described below and are summarized in Figure 1.

### 2.1. Fixation of Cells

The first and most critical step for the visualization of biological objects by EM is the fixation, because it determines how closely the image seen in the microscope resembles the *in vivo* structure. The aim is avoiding or reducing artifacts caused by extraction, denaturation, steric hindrance, chemical alteration of epitopes (especially important for immunocytochemistry analysis) and changes in volume and shape (critical for the 3D analysis methods described in this review) [14]. Fixation of cells can be carried out by two different methods: chemical fixation (Figure 1A) or cryo-immobilization (*i.e.*, physical-fixation; Figure 1B).

#### 2.1.1. Chemical Fixation

Chemical fixation of cells is usually performed with buffered aldehydes. During this step the aldehydes create an inter- and intra-molecular network of covalent interactions (“cross-links”), mostly between amino groups that stabilize the biological sample. This results in the formation of a large 3D network of irreversible cross-links throughout the cytoplasm in tenths of seconds to minutes.

While most laboratories have their own preference, glutaraldehyde (GA) either alone or in combination with paraformaldehyde (PFA) are the most commonly used primary fixatives. However, GA induces a branched meshwork of cross-links that sterically hinder accessibility of antibodies to the antigen. For this reason, formaldehyde, which masks less the antigenicity, is mostly used for immunocytochemistry studies like immunofluorescence [14].

During all the processing steps it is very important that cells do not dry out. This is particularly important before fixation, prior to the initial contact with the fixative, when the cells are still alive. Although the fixation process kills cells, cells should die “properly” to ensure that, as far as possible, all cell components are kept so well preserved as when they were alive. To this aim, several other factors might be taken into consideration. Hence the purity of the aldehydes is also critical. Therefore it is recommended to use EM grade aldehydes provided by commercial suppliers. Furthermore, the cross-linking ability of these aldehydes is influenced by the time, concentration and temperature [19]. Routine fixation is performed during 15–30 min at room temperature using a buffer with a physiological pH (6.8–7.4) and a concentration of at least 0.1 M (mol/L) [14]. The nature of the buffer plays also a very important role during fixation: sodium cacodylate, sodium phosphate, HEPES (4-(2-hydroxyethyl)-1-piperazineethanesulfonic acid) and PIPES (piperazine-N,N′-bis(2-ethanesulfonic acid)) are, for instance, among the most widely used.

Unfortunately there is not a general recipe for chemical fixation. The best results require adaptations to individual experimental conditions. Therefore, we recommend consulting an EM specialist or check the literature to help you to choose the optimal conditions (including type and concentration of the aldehyde; type, concentration and pH of the buffer; time and temperature for fixing), tailored for your experiments.

**Figure 2:**
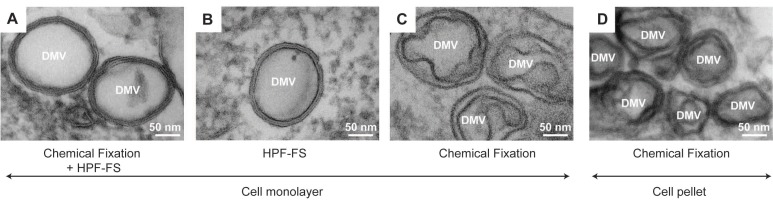
Impact of different fixation methods on the morphology of double membrane vesicles (DMVs) induced by Hepatitis C Virus (HCV). (**A**) Huh7 cells grown on sapphire discs were infected with HCV and processed after chemical fixation by high pressure freezing (HPF) and freeze substitution (FS); (**B**) Huh7 cells were transfected by electroporation with a subgenomic HCV replicon RNA and 48 h later subjected directly to HPF-FS without chemical fixation. DMVs were also found in high abundance in these samples excluding that they are an artifact caused by chemical fixation. Also note the minimal extraction of the cytosol surrounding the DMVs and their content in comparison to chemically fixed cells; (**C**) HCV-infected Huh7.5 cells grown on coverslips were chemically fixed and then embedded in epon; (**D**) Huh7.5 cells were infected with HCV and 48 h later cells were fixed, scraped off the culture dish and sedimented by gentle centrifugation prior to embedding of the cell pellet in epon resin. Note the “fried-egg”-like morphology of the DMVs observed in chemically fixed cells (**C**,**D**), in comparison to the very well-delineated membranes of cells subjected to HPF (**A**,**B**). EM micrographs were taken with permission from Romero-Brey *et al.* [20].

Upon fixation cells are washed with the buffer of choice, in which cells can be stored at 4 °C until further processing. Note that cultured cells can be prepared for EM as monolayers or as pellets (Figure 1A). When the cells are further processed as pellets, they must be scraped off the cell culture dish after fixation if they are not growing in suspension. Alternatively, for cryo-EM cultured cells can be “trypsinized” before fixation (Figure 1B). It should be kept in mind that pelleting of the cells alters their morphology and can lead to artifacts (Figure 2). Therefore -whenever possible- we highly recommend the use of cell monolayers grown on flat supports such as coverslips (to be used for chemical fixation; Figure 2A,B), sapphire discs (for high pressure freezing; Figure 2C) or on EM grids (for cryo-EM; Figure 1).

The main drawback of chemical fixation is that it alters the structure of the cell by forming a network of cross-linked molecules. Such a network is prone to artifacts, which is a major challenge for most EM-based studies (for a detailed discussion on this topic see [21]). Nevertheless, chemical fixation has been a mainstay of EM for decades as it preserves the cell morphology reasonably well. Indeed Dubochet and others [22,23] have shown that the major organelles of well-studied cells look essentially the same in chemically and non-chemically fixed cells. Along these lines, the overall appearance of the Hepatitis C Virus (HCV)-induced double membrane vesicles (DMVs) is strikingly similar between specimens prepared with different methods (Figure 2) [20].

As already pointed out by Small 34 years ago [24], the majority of ultrastructural alterations might occur during the post-fixation processing of the samples for embedding (described below), rather than during fixation. Thus, a tailored protocol must be designed for the highest preservation of cells/tissue of interest, including both optimal fixation and post-fixation conditions.

#### 2.1.2. Cryo-Fixation

Freezing techniques represent an alternative to the artifact-prone chemical fixation (reviewed in [25]). The basic principle is to arrest cells by rapid cooling, a process that takes a few milliseconds, resulting in the simultaneous stabilization of all cellular components without altering their environment. The simplest method consists of immersing cells growing on EM grids in liquid ethane or propane, by means of plunge [26,27] and jet freezing [28,29,30,31,32], respectively (Figure 1B). An inherent limitation of these rapid cooling approaches is that samples can only be vitrified to a depth of micrometers from their surface [33,34,35]. This lies in the poor heat conductance of water: high superficial cooling rates rapidly decay within the sample, reaching a low value that causes water crystallization [36,37,38]. Ice crystals alter the cytoplasm ultrastructure by inducing phase segregation between water and solutes [37,39]. Even worse, growing ice crystals might lead to the formation of holes in membranes and destroy organelles [40].

A way of preventing ice formation is pre-incubating the biological samples with anti-freeze agents to reduce the concentration of free water, such as sucrose, glycerol, DMSO or various polymers. However, the use of these cryoprotectants introduce alterations in the original cytoarchitecture [40]. Thus, the unique means to preserve cells in its native state in absence of cryoprotectants is to freeze them in such a way that the water of the living cells turn into vitreous ice [37]. This can be achieved by high pressure freezing (HPF) [41], with which the vitrification depth can be increased more than 10-fold (up to 200 µm) in comparison with plunge and jet freezing ([38,42], reviewed in [43]). Using high pressure (~2000 bar) prevents the expansion of water, lowering its freezing point, increasing the freezing rate and reducing the crystallization rate of ice (reviewed in [25]). Note that, however, for highly hydrated tissues or cell suspensions, vitrification may require indeed the use of cryoprotectans prior to high pressure freezing (e.g., soaking the samples in a non-penetrating cryoprotectant such as 20% dextran (w/v); [44]).

In the case of HPF, owing to the high pressures used for freezing, cells must be grown on resistant supports like sapphire [45,46] or aclar [47,48,49] discs (Figure 1B). Sapphire discs are normally carbon coated to improve cell attachment and this carbon coat serves as a predetermined breaking layer after sample embedding into resin [50]. Sapphire/aclar discs can be subsequently frozen by assembling them between two aluminium planchettes. Pelleted cell suspensions resuspended in e.g., dextran, can be also placed between two planchettes for freezing. Alternatively pellets can be frozen in small tubes as described by Hohenberg *et al.* [51], where the cells are loaded by capillary forces.

#### 2.1.3. Combination of Chemical and Cryo-Fixation

Handling of infectious specimens such as viruses requires working under strict biosafety level (BSL) conditions, which is particularly important when working with human samples. Since equipment for sample preparation and EM analysis is rarely available in high containment biosafety laboratories, samples must be inactivated before leaving these facilities, which is achieved by chemical fixation. Note, however, that few BSL laboratories in the world are equipped with freezing devices, as well as with cryo-microscopes (e.g., [52]), where examination of virus-infected cells can be performed in their most genuine state. Chemically inactivated samples can be also subsequently subjected to cryo-fixation outside the BSL laboratory. Although this double-fixation sounds redundant, as reported for the Flock House Virus (FHV) [53] this combination can result in an even better preservation of the subcellular structure as compared to samples preserved by chemical fixation alone. Along the same lines, we have also recently shown that this fixation technique leads to an excellent preservation of the DMVs induced by HCV [20] (Figure 2). In fact, GA fixation prior to HPF (Figure 2A) resulted in a preservation of the DMVs close to that found in cryo-fixed cells (Figure 2B) and was superior to that obtained by conventional chemical fixation alone (Figure 2C,D). Note that this hybrid method provides a more near-to-native morphology of the DMVs, depicting well-delineated membranes, compared to the “fried-egg”-like DMVs observed within chemically fixed cells. It is important to bear in mind, however, that this “fried-egg” appearance might be due to the performance of the dehydration at room temperature, instead of at 4 °C, that it has been shown to reduce the loss of lipids [15] and might, therefore, result in attaining smoother membranes. This alternative dehydration protocol has been successfully used, for instance, to study the Rubella Virus factories [54]. In our hands this hybrid method also caused a slight extraction of the cytosol, thus enhancing visibility of the membrane layers (Figure 2A). For all these reasons, we recommend to use the combination of these methods rather than chemical fixation alone. However, a note of caution should be added that aldehyde pre-fixation might change the morphology of some cell organelles as previously reported [55].

### 2.2. Embedding of Cells

#### 2.2.1. Embedding of Chemically Fixed Cells

After chemical fixation, cells must be further processed in order to analyze them by EM. Due to their low electron scattering power biological samples are inherently of low contrast [56]. Therefore, heavy metals like osmium tetroxide (OsO_4_) and uranyl acetate (UA), with high affinity to many cellular structures, are used after fixation as contrasting agents in routine EM. Owing to its reactivity with unsaturated acyl chains of membrane lipids OsO_4_, for instance, facilitates the retention of lipids [57,58], in addition to its role in contrasting structures (especially membranes). Similarly, Silva *et al.* [59] have shown that UA plays a role in protecting lipids against solvent extraction. Furthermore, OsO_4_ also acts as a protein fixative [60]. However, when used at room temperature acts more like a protease [61]. For this reason it is highly recommendable to post-fix the samples with low concentration of OsO_4_ on ice (at 0 °C) [61]. Note also that due to the harmful effect of OsO_4_ on antigens [14], osmicated cells can still be used for subsequent immunocytochemical approaches [62], but it is not the first choice.

Subsequently cells are embedded in resins. Conventional plastic embedding requires resins with heat-induced (above 50 °C) polymerization such as epoxy resins, which are often used since their first introduction in the 1950s [63]. Due to their extremely hydrophobic nature cells/tissues must be completely dehydrated in a series of ascending protein denaturing solvents (e.g., ethanol) before infiltration (Figure 1A). The resulting high degree of covalent interaction between epoxy resins and the biological material make these resins not well compatible for immunocytochemistry. Nevertheless, owing to their fine structural preservation epoxy resins are an excellent embedding medium for high-resolution structural preservation and 3D analysis.

#### 2.2.2. Embedding of Cryo-Fixed Cells

After cryo-immobilization, cells can be stored in liquid nitrogen (LN_2_) or immediately further processed by means of freeze substitution (FS) (Figure 1B) (recently reviewed in [64]). In this case, frozen cells are put into a mixture of cross-linking chemicals (such as aldehydes, OsO_4_, UA or tannic acid) with an organic solvent (acetone, ethanol or methanol) at temperatures around −90 °C [65,66]. It has been also reported that membrane visibility can be improved by adding 1%–5% water to the substitution medium [67].

Subsequently the embedding medium is introduced with the solvent, while the temperature is allowed to increase gradually. The initial polymerization of the resin is usually carried out at sub-zero temperatures. The idea behind this is that low temperatures tend to stabilize proteins during the removal of the solvent (*i.e.*, water) minimizing putative dehydration effects [68] and thereby protecting proteins from denaturation. For this reason low viscosity resins (e.g., Lowicryl, LRWhite and LRGold resins) which enable infiltration at very low temperatures, are mostly used. These resins also have a low tendency to co-polymerize with the sample structure [69] and are able to preserve the fluorescence, and are therefore, mostly used for immunocytochemistry [70,71] and correlative light electron microscopy (CLEM) [72,73] studies. After complete infiltration of the resin, the final polymerization step can be induced at room temperature with UV light (360 nm).

Nonetheless, epoxy resins can be also used to embed freeze-substituted samples. In this case, the samples should be further dehydrated at room temperature after the substitution to be subsequently embedded and polymerized at high temperatures. In any case, long periods of infiltration are characteristic of the FS approach, which might be a practical disadvantage. However, shorter substitutions can be also carried out with satisfying results [67,74,75].

For a comparative study about the use of different resins to embed cells for 3D-EM, please see a very recent report from Bruno Humbel’s laboratory [76].

### 2.3. Sectioning of Resin-Embedded Cells

**Figure 3:**
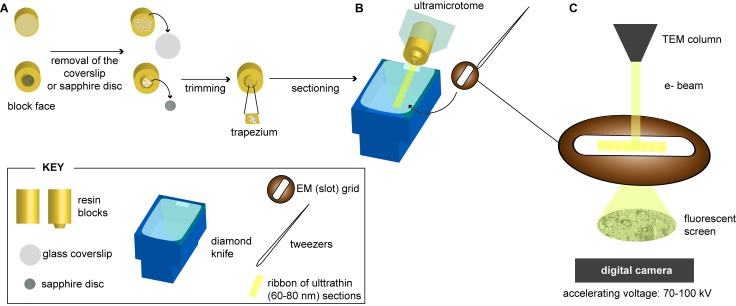
Schematic representation depicting the post-processing steps prior to the analysis of virus-infected cells by conventional-EM (“plastic-EM”). (**A**) Virus-infected cells embedded into a plastic resin are sectioned with an ultramicrotome: the resin block containing the embedded virus-infected cells must be trimmed to a trapezium-like shape with an average size of approximately 200 × 250 μm prior to its sectioning with a diamond knife equipped with a water shank; (**B**) The obtained ultrathin sections (60–80 nm, in yellow) float on the water surface from where the section ribbon can be mounted onto a formvar-coated EM grid; (**C**) After contrasting with heavy metals, the cell sections can be examined with a conventional transmission electron microscope (TEM) operated at accelerating voltages between 70 and 100 kV. In brief, an electron beam is generated (by a thermionic or a field emission gun) and accelerated under vacuum. The electrons are then transmitted through the cell sections. After passing through the specimen, scattered electrons are focused by electromagnetic lenses and magnified onto an imaging device such as a fluorescent screen or recorded with a digital camera (CCD, charged-coupled device, or a CMOS, complementary metal-oxide semiconductor). The diagram of the TEM-working principle shown on the right is adapted from Briggman and Bock [78].

In order to be analyzed by TEM, resin-embedded cells must be sectioned (Figure 3). This is due to the inability of the electrons to penetrate the entire embedded cell. To this aim the block face containing the embedded cells must be trimmed to a smaller area (regularly ~200 × 250 µm; note, however, that the size of the section is limited by the size of the 3.05 mm round standard EM grid and, therefore, the trimming could be made for a larger area when needed), which can then be sectioned with a diamond knife at an angle of 35° to minimize squeezing artifacts. Routine ultrathin sections (60–80 nm) are obtained and remain floating on the surface of the water shank of the diamond knife, from where they are collected onto an EM grid. Note that after acquiring the sections, they normally need to be contrasted before they can be visualized by TEM. The most commonly used methodology consists of post-staining grids with UA and lead salts [77]. However, when using the FS cocktail proposed by Walther and Ziegler [67], due to their higher contrast, sections can be directly observed by TEM, without requirement for on-section staining.

### 2.4. Resin-Free Processing of Cells

As stated before, the ultrastructural preservation of the cells is not only influenced by the method of fixation, but also by the subsequent processing steps (dehydration, infiltration and resin polymerization). As a consequence of the denaturing effects of these processing steps the structures are rarely preserved to the extent as they appear *in vivo*. To avoid these artifact-prone preparation procedures, the method of choice is the freezing or vitrification of the cells (described above) followed by their direct examination at very low temperatures by cryo-EM. However, due again to the limited penetration power of electrons, only the thin cell periphery of frozen cells can be visualized by standard cryo-EM [79].

Alternatively frozen cells must be sectioned in order to be observed by TEM. Hence frozen-hydrated sections can be analyzed by the so-called cryo-electron microscopy of vitreous sections (CEMOVIS). In CEMOVIS high pressure-frozen specimens (Figure 1B) are cut into ultrathin sections, as described below for resin-embedded specimens (Figure 3), but at temperatures below −150 °C (below the de-vitrification temperature to avoid sample damage). This technique was first started with the work of Fernandez-Moran [80] and was further improved among others by Dubochet and coworkers [22,27,81], Frederik and coworkers [82,83,84] and Sitte [85]. More recently improvement of cryo-microtomy techniques have resulted in CEMOVIS as a much more accessible approach ([86], reviewed in [87]), despite of its critical challenging aspects: obtaining good quality sections (without knife marks, crevasses or ripples, produced during sectioning) that remain attached to the EM grid [88] and its subsequent imaging at low temperatures and also low electron doses.

## 3. Methods to Study the 3D Architecture of Virus-Infected Cells

Obviously, the information that we can obtain from ultrathin sections of a certain region of interest (ROI) of an infected cell is limited. Since ultrathin sections with a common average size of 200 × 250 μm contain normally the profiles of several cross-sectioned cells, this limitation can be overcome even by conventional TEM when analyzing intracellular events that occur with high frequency. Moreover, even in case of relatively low rates of infection it is possible to find infected cells among these cell profiles when the virus-induced phenotype is rather striking and known. However, interpreting the 3D organization of a structure from such 2D sections is difficult and most often leads to a “simplification” of much more complex structures [89]. Furthermore, depending how these objects are sectioned conflicting interpretations can arise [90]. Thus, for a correct interpretation of a 3D structure all components must be recognizable in the section [14]. Therefore, gaining access to the ultrastructural information contained in thick sections or – ideally- in the whole volume of an infected cell is essential.

**Figure 4:**
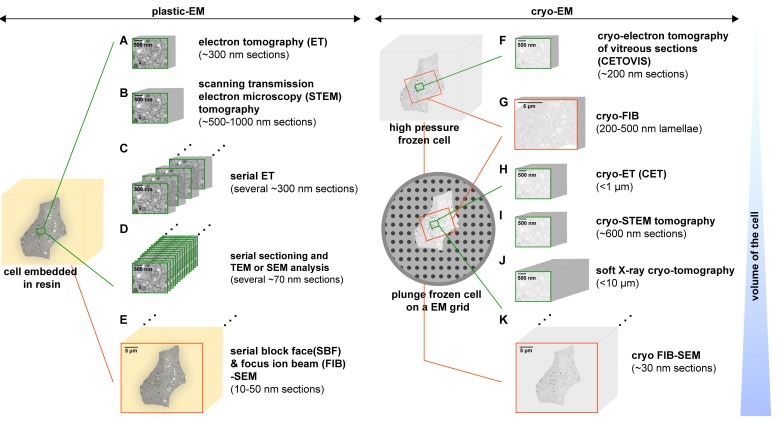
Overview of the 3D-EM methods that can be used to analyze the architecture of virus-infected cells by means of plastic-EM (A-E, on the left) or cryo-EM (F-K, on the right). (**A**,**B**) Thicker sections of approximately 300 nm and 500–1000 nm of a region of interest (ROI, highlighted in green) can be analyzed by ET and Scanning Transmission Electron Microscopy (STEM) tomography, respectively; (**C**) Several sequential tomograms can be also joined together to reconstruct a larger volume of the ROI; (**D**) Manually obtained serial ultrathin sections of a ROI can be imaged by conventional TEM and the information of these 2D images combined to create a 3D map of this particular area. Alternatively, sectioned cells can be analyzed by Scanning Electron Microscopy (SEM); (**E**) With serial block face (SBF) or focus ion beam (FIB)-SEM 3D information of a whole virus-infected cell (framed in orange) can be obtained; (**F**) Alternatively, ~200 nm sections of high-pressure frozen cells can be further analyzed at very low temperatures by CETOVIS (cryo-electron tomography of vitreous sections); (**G**) A FIB can be also used to slice 200–500 nm thick lamellae from vitrified (high pressure or plunge frozen) cells that can be then analyzed by cryo-ET; (**H**) Cryo-ET (CET) can be applied to entire plunge frozen cells. However, it only allows getting information from where the cell thickness is ≤1 µm (at the cell periphery); (**I**) Cryo-STEM tomography can be used to study ~600 nm thick sections of vitrified cells (at 200 kV); (**J**) With soft X-ray cryo-tomography 3D information of up to 10 µm can be retrieved from frozen hydrated specimens; (**K**) A FIB can be also used to slice 30 nm sections from the block face of vitrified cells that can be then recorded in a sequential fashion by SEM. Note that techniques allowing for wider fields of view are highlighted in orange, whereas in green are those allowing the imaging of smaller fields of view.

Several approaches have been developed to gain 3D information of cellular structures by EM (Figure 4). In the following sections these approaches and their applications to study viral infection (summarized in Table 1) are described.

**Table 1:** Summary of the EM methods used to reconstruct the 3D architecture of virus-infected cells.

3D-EM Method	Virus	Cells *	Step of the Virus Life Cycle Analyzed	Reference
**Electron Tomography (ET)**	Flock House Virus (FHV)	Drosophila S2	Replication	[91]
	Herpes Simplex Virus type 1 (HSV-1)	HEp-2	Assembly (Envelopment)	[92]
	Rice Dwarf Virus (RDV)	Leafhopper vector	Release	[93]
	Human Immunodeficiency Virus-1 (HIV)-1 & Simian Immunodeficiency Virus (SIV)	T cells	Entry	[94]
	SARS-Coronavirus	Vero E6	Replication	[95]
	HIV-1	MT-4 & HeLa	Assembly & Budding	[96]
	Human T-Lymphotropic Virus 1 (HTLV-1)	MS9 & Jurkat	Spread	[97]
	Vaccinia Virus (VV)	HeLa	Assembly	[98]
	Dengue Virus (DENV)	Huh-7	Replication & Assembly	[99]
	VV	HeLa spinner	Assembly	[100]
	Rice Gall Dwarf Virus (RGDV)	VCM	Release	[101]
	Murine Gammaherpesvirus	NIH 3T3	Attachment, Entry, Assembly & Egress	[102]
	West Nile Virus (WNV)	Vero	Replication	[103]
	DENV	C6/36, Vero & SK Hep1	Replication	[104]
	SARS-Coronavirus	Vero E6	Replication	[105]
	Rubella Virus	BHK-21	Replication	[54]
	Marburg Virus (MARV)	Huh-7	Budding & Release	[106]
	Semliki Forest Virus (SFV)	BHK-21	Budding	[107]
	Coxsackievirus B3 (CVB3)	Vero E6	Replication	[108]
	RGDV	NC-24	Replication	[109]
	Equine Arterivirus (EAV)	Vero E6	Replication	[110]
	Poliovirus type I	HeLa	Replication	[111]
	Hepatitis C Virus (HCV)	Huh 7.5	Replication	[20]
	Langat Virus	Vero & ISE6	Replication & Assembly	[112]
	Tick Borne Encephalitis Virus (TBEV)	BHK-21	Replication & Assembly	[113]
	Infectious Bronchitis Virus (IBV)	CK	Replication	[114]
	VV & Mimivirus	HeLa & *A. polyphaga*	Assembly	[115]
	WNV	BHK	Assembly	[116]
	DENV	C6/36	Replication & Assembly	[117]
	Beet Black Scorch Virus (BBSV)	*Nicotiana benthamiana*	Replication	[118]
	HIV-1	Gut-associated lymphoid tissue	Budding, Release & Cell-to-cell transmission	[119]
	WNV	Vero	Replication & Assembly	[120]
	*Autographa californica* Multiple Nucleopolyhedrovirus (AcMNPV)	Sf9	Assembly (Envelopment)	[121]
	TBEV	HN	Replication & Assembly	[122]
	African Swine Fever Virus (ASFV)	COS	Replication & Assembly	[123]
**Scanning Transmission Electron Microscopy (STEM) tomography**	Mimivirus	*Acanthamoeba polyphaga*	Replication & Assembly	[124]
	ASFV	COS	Replication & Assembly	[123]
	Paramecium Bursaria Chlorella Virus 1 (PBCV-1)	*Chlorella variabilis*	Replication & Assembly	[125]
**Serial Sectioning**	VV	PtK2	Replication & Assembly	[90]
	Bunyavirus	BHK-21	Replication & Assembly	[126]
	Varicella-Zoster Virus (VZV)	MeWo	Assembly	[127]
	HCV	Huh 7.5	Replication & Assembly	[128]
	Human Cytomegalovirus –(HCMV)	Fibroblasts, human endothelial cells & macrophages	Assembly (Envelopment)	[50]
	Reovirus	HeLa	Assembly	[129]
	Zucchini Yellow Mosaic Virus (ZYMV)	*Cucurbita pepo* L. plant cells	Spread	[130]
**Focus Ion Beam-Scanning Electron Microscopy (FIB-SEM)**	HIV-1	Macrophages	Release	[131]
	HIV-1	T & dendritic cells	Cell-to-cell transmission	[132]
	HIV-1	T cells & astrocytes	Cell-to-cell transmission	[133]
	PBCV-1	*Chlorella variabilis*	Replication & Assembly	[125]
**Cryo-ET (CET)**	VV	Ptk2	Disassembly	[134]
	HSV-1	Vero, PtK2, and HFF	Entry	[135]
	HIV-1	MDM	Release	[131]
	HIV-1	U-87 MG and U-373 MG	Assembly & Budding	[136]
	MARV	Huh-7	Assembly & Budding	[137]
	HSV-1	Hippocampal neurons	Assembly (Envelopment)	[138]
	Influenza A Virus	MDCK	Budding	[139]
	Bacteriophage T7	*Escherichia coli*	Entry	[140]
	RDV	NC24	Egress	[141]
	Syn5 Cyanophage	WH8109	Assembly	[142]
	Baculovirus	B16	Spread	[143]
	HIV-1	HUVEC	Release	[144]
**Soft X-ray Cryo Tomography**	VV	PtK2	Replication & Assembly	[90]
	Pseudorabies Virus (PrV)	EFN-R	Assembly (Envelopment)	[145]

* For a detailed description of the cell types please refer to the original publications. Plastic-EM methods are shown on the top, and cryo-EM methods on the bottom.

### 3.1. Electron Tomography (ET)

#### 3.1.1. Description

**Figure 5:**
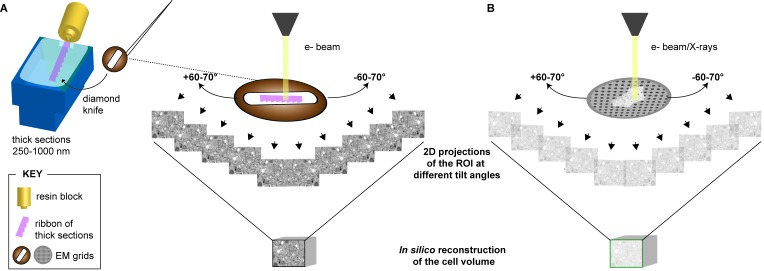
Principle of electron tomography (ET) and 3D reconstruction. (**A**) Thicker sections (250–1000 nm, in pink) of virus-infected cells can be analyzed by means of electron tomography (ET) or scanning transmission electron microscopy (STEM) tomography using microscopes operated with voltages >100 kV. The sections are tilted relative to the incident electron beam in an automatic fashion with help of a goniometer. 2D projections of the same field of view are acquired every tilted angle, from which the cell volume is subsequently reconstructed *in silico*; (**B**) Following the same working principle, cryo-ET, cryo-STEM tomography or soft X-ray cryo-tomography can be applied to vitrified cells grown on EM grids. For this purpose, the microscope must be operated at very low temperatures and with low electron doses (in the case of cryo-ET and cryo-STEM tomography). Alternatively thick cryo-sections collected on an EM grid as in (**A**) can be analyzed by CETOVIS (see text for further details). The diagrams of the ET-working principle are adapted from Baumeister *et al.* [147].

The thickness of the section of virus-infected cells is a limiting factor, especially in case of small structures such as virions that are often difficult to find. To overcome this problem thicker sections (~300 nm) can be analyzed by means of electron tomography (ET) ([146,147,148]) (Figure 4A and Figure 5).

ET was first used to gain structural information on viral particles [149,150]. It is based on the principle defined by Radon in his seminal paper of 1917 [151]: 3D information (a tomogram) can be retrieved from the projections of an object. The penetration depth of electrons through an object is directly correlated to the energy of the electrons and thus, the higher their accelerating voltage, the thicker the objects that can be analyzed [152]. Therefore the acquisition of tomograms requires not only a microscope equipped with a goniometer (that allows tilting the EM grid containing the sections by 60°–70° in either direction), but also requires high voltage microscopes (operated at 200–300 kV) (Figure 5). The 2D projections of the objects obtained at different tilt angles are then reconstructed *in silico* to retrieve the 3D volume of the cell (Figure 5). Tilting the specimen in only one orthogonal axis (single-axis tomography) results in the so-called missing-wedge information. To partially overcome this problem the specimen can be tilted around two orthogonal axes (dual-axis tomography). The two tomograms are then computed from each tilt series and combined with general 3D linear transformations that can correct for distortions between the two tomograms [153,154]. Although this method does not allow gaining access from all the possible tilt angles, it reduces at least the missing-wedge to a missing-pyramid, resulting in a gain of 3D information.

Despite of being a very powerful and widely used technique, the information obtained through ET is from a small percentage of the whole cell volume. To obtain information from larger volumes, tomograms from consecutive sections can be joined (serial ET) [155,156] (Figure 4C). However, due to compression happening during sectioning or material collapse occurring during electron beam exposure [157], which might lead to a loss of information within and between sections, reconstruction of a large volume from several tomograms is challenging. Other approaches may be used instead (see below).

#### 3.1.2. Applications to the Study of Virus-Infected Cells

**Figure 6:**
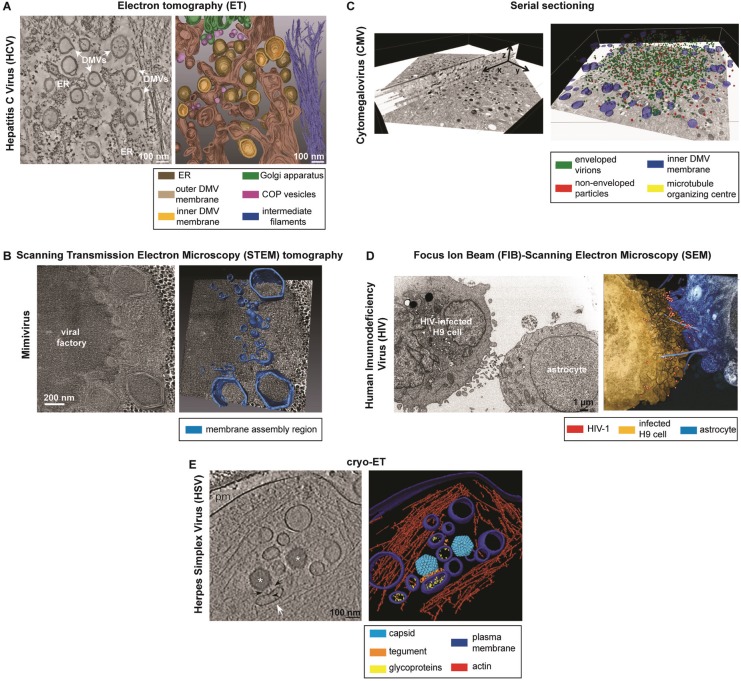
Examples of applications of 3D-EM methods to the study of human-relevant viral infections. (**A**) Electron tomography (ET) of Hepatitis C Virus (HCV)-infected cells [20]. On the left: slice of a tomogram showing HCV-induced double membrane vesicles (DMVs); on the right: 3D surface rendering of the whole tomogram, showing DMVs in close proximity to ER; (**B**) STEM tomography of Mimivirus-infected cells [124]. On the left: digital slice derived from STEM tomography of 280 nm thick section; on the right: 3D surface rendering revealing that the membrane assembly region consists of an elaborate membrane network; (**C**) Serial sectioning of cytomegalovirus (CMV)-infected cells [50]. On the left: single micrograph (in X–Y direction) and image stack (slice through the Z-axis) of the assembly complex; on the right: cellular and viral structures were segmented on all 28 sections and superimposed on a single micrograph; (**D**) FIB-SEM of HIV-infected cells [133]. On the left: 2D FIB-SEM image of an HIV-1 chronically infected H9 cell (left) and an uninfected astrocyte (right), co-cultured for 24 h; on the right: 3D rendering of the FIB-SEM image stack containing the contact zone between the HIV-infected H9 cell and the astrocyte. The target cell extends long filopodial bridges towards the infected cell across the intercellular gap. HIV-1 virions are detected adjacent to the filopodial bridges; (**E**) Cryo-ET of Herpes Simplex Virus (HSV)-infected cells [138]. On the left: slice of the tomogram, showing secondary envelopment of capsids; on the right: 3D surface rendering of the whole tomogram, showing a capsid in close proximity to an enveloping vesicle. Asterisks: capsids; white arrow: enveloping vesicle; arrowhead: glycoproteins; black arrows: tegument; pm: plasma membrane. All these pictures are reproduced from the original publications with permission (see Acknowledgments).

ET has become an important method for virologists and was used e.g., to visualize the remodeling of intracellular organelles and the distribution of virus particles within the host cell (reviewed in [158]). In fact, most of the literature pertains to the use of this approach (Table 1). The bulk of these studies relates to virus-induced remodeling of cell membranes in order to build up their replication organelles, mostly positive-strand RNA viruses (reviewed in [159]). An example of the ET analysis of HCV-induced replication factories is shown below (Figure 6A).

### 3.2. Scanning Transmission Electron Microscopy (STEM) Tomography

#### 3.2.1. Description

Apart from serial ET, another way of circumventing the limited thickness of ET is the use of Scanning Transmission Electron Microscopy (STEM) allowing the collection of tomographic datasets (Figure 4 and Figure 5). The main advantage of using the STEM modus is its scanning geometry that allows for dynamic focusing so that the imaging conditions are uniform across a tilted specimen [160]. Furthermore, its use for tomography of thick, plastic-embedded sections has revealed an improved signal-to-noise ratio (SNR) and far higher contrast over conventional TEM tomography [161,162,163,164].

By means of STEM tomography specimens up to 1 μm can be examined [164,165,166] (Figure 4B). However, since the total thickness of a cell is 10–20 µm or more, the information retrieved from 1 μm thick sections is still quite limited.

#### 3.2.2. Applications to the Study of Virus-Infected Cells

This approach is ideal for the study of large cellular organelles or viruses, which size exceeds the thickness achieved by conventional ET. Thus, STEM tomography has been applied to the study of large dsDNA viruses, like the giant Mimivirus [124] (Figure 6B, Table 1), as well as to dissect the 3D architecture of the viral factories induced by African Swine Fever Virus (ASFV) [123] or Paramecium Bursaria Chlorella Virus 1 (PBCV-1) [125] (Table 1).

### 3.3. Serial Sectioning

#### 3.3.1. Description

**Figure 7:**
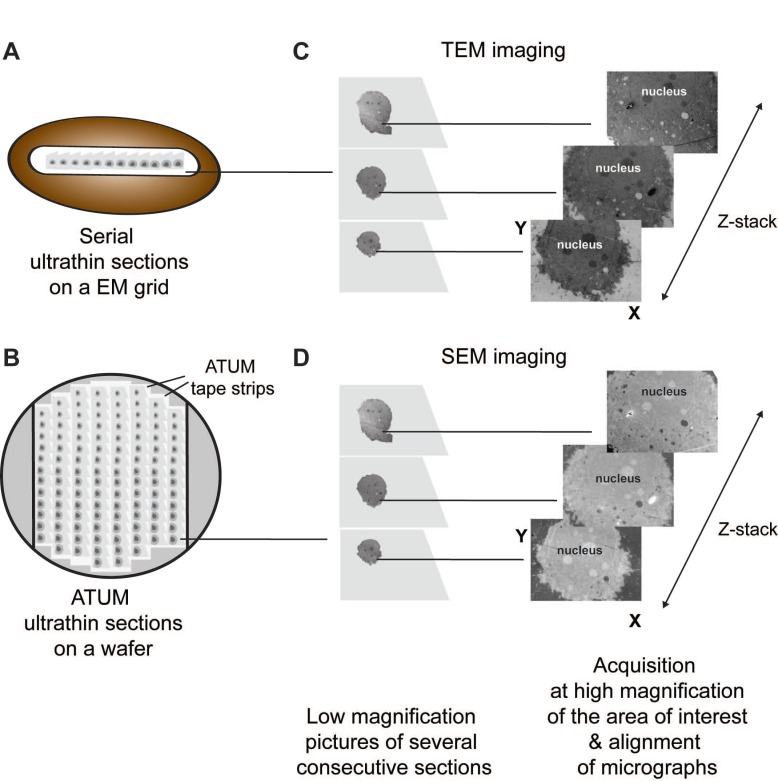
Serial sectioning workflow. A ribbon of consecutive ultrathin sections (60–80 nm) of a cell can be either obtained manually (**A**) or automatically on a tape (ATUM, automatic tape-collecting ultramicrotome) (**B**) and subsequently analyzed by TEM (**C**) or SEM (**D**), respectively. Generated micrographs are aligned to obtain a z-stack of this cell. Note also that the same principle is used to join tomograms obtained by serial-ET.

The thickness limitations of the above-described methods can be overcome by analyzing consecutive thin sections (serial sections) of cells. Serial sectioning, in use since 1958 [167], can be performed to the entire cell volume and provide, therefore, information about its architecture. It has the main advantage that it does not need special equipment, being practicable at any EM facility (Figure 4D). However it requires well-trained personnel and patience to acquire a long ribbon of sections of the cell of interest, without losing a single section. Micrographs obtained either by TEM or scanning EM (SEM) must be taken from every section and be aligned to obtain a stack of images (Figure 7). This technique can be also applied to study the anatomy of relatively large organisms like nematodes. Hence, as a result of the reconstruction of about 8000 or more than 3000 micrographs [168,169], respectively, the structure of the worm nervous system was successfully attained.

A similar method, called array tomography has been developed in which arrays of serial ultrathin sections collected in glass slides are first fluorescently labeled (with antibodies or stains) and subsequently imaged by confocal microscopy to get a 3D distribution of antigens [170]. Interestingly the array can be repeatedly eluted, re-stained and imaged again to assess the distribution of other antigens. Finally it can be stained with heavy metals and analyzed under a SEM microscope. This combination method allows the correlation of volumetric imaging of antigens with ultrastructural imaging.

The main drawback of this technique, however, is discontinuities between two consecutive sections due to compression artifacts generated by the sectioning or more dramatically the loss of sections. Furthermore, these distortions of sections occurring during cutting, staining and imaging [171,172] hinder the subsequent alignment step, resulting in a poor resolution in the Z-plane.

To avoid the laborious and prone to errors manual sectioning, sections can be collected automatically for SEM imaging on electron-opaque plastic tapes (ATUM, automatic tape-collecting ultramicrotome) [173]. Subsequently images of ATUM sections can be also taken in an automatic manner [174], allowing for high throughput image acquisition. ATUM-SEM allows to reliably cut thousands of sections as thin as 30 nm.

#### 3.3.2. Applications to the Study of Virus-Infected Cells

Although labour intensive, manual serial sectioning has been used to study virus-infected cells by means of TEM (described in [175,176]), including vaccinia virus, bunyavirus, HCV, cytomegalovirus, reovirus and a plant virus (Table 1). Thus, the distribution of envelopment events within the human cytomegalovirus (HCMV)-induced assembly complexes could be visualized using this technique [50]. To this aim a total of 28 sections with a thickness of 100 nm were imaged, leading to a total volume of 2.8 μm (Figure 6C). Another example relates to a plant virus [130]: 105 sections (with 80 nm thickness; a total of 8.4 μm) of cells containing cylindrical inclusions (CIs) revealed that these structures form long tubes throughout the cytosol.

Furthermore, serial sections of Varicella-zoster (VZV)-infected cells have been analyzed by SEM [127], which allowed the reconstruction of a total nuclear volume of 291 µm^3^ from a ribbon of 82 consecutive serial sections with a 100 nm thickness.

### 3.4. Serial Block Face (SBF) and Focus Ion Beam (FIB)-Scanning Electron Microscopy (SEM)

#### 3.4.1. Description

**Figure 8:**
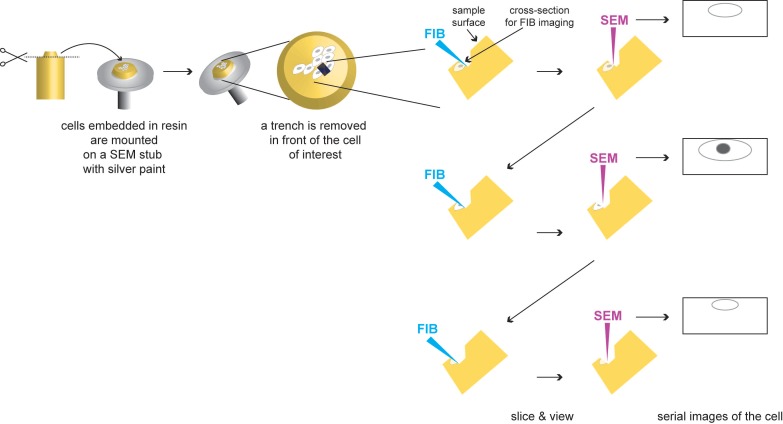
Principle of Focus Ion Beam (FIB)-Scanning Electron Microscopy (SEM). Epon blocks containing the embedded cells are mounted on SEM stubs with silver paint. First a trench is milled with the FIB, generating a cross-section into the epon block. Subsequently the newly generated block face is milled (with the FIB, blue), resulting in the removal of a thin layer (as small as 3 nm; [181]) and imaged (with the SEM, pink) in a sequential manner. This process is repeated automatically as long as needed to obtain a tomographic dataset. Further details about this method can be found in the main text.

Creating reconstructions of cells from sections whose thicknesses exceed the limit of the penetrating power of TEM can be overcome by the use of a scanning electron microscope (SEM) to collect information of the block face upon removal of very thin sections (Figure 4E). The working principle of these approaches is the same as with serial sectioning. However, in contrast to serial sectioning, with Serial Block Face-SEM (SBEM, formerly called SBF-SEM by W. Denk [177]) and Focus Ion Beam (FIB)-SEM (Figure 8) the sectioning is integrated inside the SEM microscope and carried out in a fully automated manner with the help of a diamond knife [177] or a focused ion beam ([178,179], and others), respectively, that act as nano-scalpels. Once a thin slice is made (3–50 nm) the block face is imaged with SEM using low accelerating voltage. In this SEM technique, backscattered electron detection is normally used to specifically view heavy metal-stained cellular components. However, it has been recently shown that the resolution of this approach is considerably improved when a secondary electron signal is used [180].

After each image is taken, a new thin slice is removed from the block face that is again imaged. The sequential “slicing and imaging” process can be repeated *ad libitum* allowing to produce a stack of images that can be computationally combined into a 3D reconstruction [182] (Figure 8). The lack of compression artifacts allows a more accurate merging of sequential images to obtain large datasets. After contrast reversal the images look similar to traditional TEM micrographs.

The cells can be prepared as for TEM. Note only that for FIB-SEM harder and more stable resins like Durcupan are recommended, to prevent damage of the sample while slicing with the powerful ion beam. An improved protocol for cell preparation to be analyzed in this way has been recently published [183]. The embedded cells must be subsequently trimmed and mounted on a SEM specimen stub with silver paint (Figure 8). Finally, due to the non-conductive nature of biological cells, the whole specimen must be platinum or gold alloy coated to improve conductivity.

In comparison with the cumbersome manual acquisition of serial sections, these approaches are far less laborious. The Z-resolution of these techniques is defined by the thickness of the slices, which represents a pitfall in the case of SBEM. However, recent developments in the FIB-SEM field allow imaging larger field of views and a section thickness of 3 nm [181] allowing the Z-resolution of FIB-SEM to approach that of ET. Furthermore, the dual beam instrument permits targeted, site-specific imaging, which makes it ideal for correlative microscopy approaches.

However, FIB-SEM has also some limitations: it requires a quite expensive specialized instrument and the sections cannot be re-examined again. Furthermore, processes like charging and electron beam damage can result in unsuccessful imaging of the surface of interest [179].

#### 3.4.2. Applications to the Study of Virus-Infected Cells

To our knowledge, SBEM has not been applied yet to the study of viral infection. However, FIB-SEM, also called Ion Abrasion (IA)-SEM, has been extensively employed for the study of HIV release and spread (Figure 6D) [131,132,133] and, more recently, also to the study of a chlorovirus [125] (Table 1).

### 3.5. Cryo-Methods

An alternative to the analysis of resin-embedded cells (plastic-EM) is the analysis of unstained, frozen-hydrated cells (cryo-EM), in which the contrast is formed exclusively by the density of the biological material itself [184,185] (Figure 4). However, vitrified specimens have low inherent contrast (that can be partially overcome with the use of better detectors) and are electron-dose sensitive. Due to these challenging aspects, cryo-EM is not as widely used as plastic-EM, but in returns yields datasets with very high resolution, disclosing molecular details of the cellular landscape.

#### 3.5.1. Cryo-Electron Tomography of Vitreous Sections (CETOVIS)

As for resin-embedded cells, thick sections of frozen cells can be collected on a grid and subsequently analyzed by ET. This technique, known as CETOVIS (cryo-electron tomography of vitreous sections) or TOVIS (tomography of vitreous sections), was first successfully used in 2002 ([186,187,188,189,190]) (Figure 4F).

Cryo-ultramicrotomy has shown its capability in producing samples of vitrified cells and tissues for visualization by cryo-TEM and tomography [191,192,193]. However, sectioning at cryogenic temperatures is notoriously difficult and artifact-prone: sections inevitably suffer from adverse distortions caused by the harsh mechanical interactions of a diamond knife and a moving specimen block during the cutting process [194]. These technical limitations have prevented both CETOVIS, but also CEMOVIS (described above) from becoming more commonly used approaches [195].

#### 3.5.2. Cryo-FIB

Cryo-FIB represents an emerging alternative technique for “thinning” frozen biological specimens without the above mentioned limitations of cryo-ultramicrotomy [196]. Thus it has been recently shown that cryo-FIB milling of frozen (both plunge and high pressure frozen) cells can produce homogeneously thin (200–500 nm), distortion free lamellae for cryo-electron tomography (CET) [197,198,199] (Figure 4G).

#### 3.5.3. Cryo-ET (CET)

CET, following the same working principle as plastic-ET, can be applied to entire vitrified cells (Figure 5B), avoiding the arduous cutting step (reviewed in [147,187,200]). CET is routinely performed with plunge frozen cells [27] (Figure 4H). Although this enables in theory the analysis of the whole cell volume, CET can only be applied to specimen areas with a maximal thickness of ~1 μm, the penetration limit of electrons [148]. Therefore though many organelles and subcellular structures can be imaged directly in plunge-frozen adherent cells, in practice it is restricted to visualize the thin edges of the cell.

After its first use in 2007 to address structural details of pathogen-host cell interaction [134], several virological studies based on this method have been reported (reviewed in [201]) (Table 1) with the herpesvirus life cycle being the most comprehensively studied [202]. One example is the study of the envelopment of Herpes Simplex Virus (HSV) particles [138] (Figure 6E).

#### 3.5.4. Cryo-STEM Tomography

STEM tomography, as described many years ago already, can be also applied to unstained vitrified specimens [203,204]. As for standard STEM tomography (described above), the increase in acceptable specimen thickness of cryo-STEM tomography (also called CSTET) broadens its applicability (Figure 4I).

Due to the weak electron scattering properties of light elements, the main components of biological, unstained vitrified specimens are, in principle, not the ideal candidates for STEM imaging. However, scattering is not so much weak as directed to low angles, and the spot scanning of STEM can be turned into an advantage regarding sample damage [205]. Thus this approach provides information from ~600 nm frozen eukaryotic cells with a microscope operated at 200 kV [206]. Working with higher voltage microscopes should then enable the analysis of even thicker samples.

As stated by Wolf and colleagues [206] apart from the shared advantages with STEM tomography of resin-embedded samples (described above), one of the main practical advantages of CSTET in comparison to CET is the lack of a need for defocusing to generate contrast, thus eliminating the ensuing complications.

#### 3.5.5. Soft X-ray Cryo-Tomography

To observe thicker samples under cryo-conditions without the need to generate sections, soft X-ray cryo-tomography can be also used ([207], reviewed in [208]). It is a powerful method that takes advantage of the high penetration power of X-rays (up to 10 µm) [209,210] without using any fixative or contrasting reagent [211,212] (Figure 4J). Its intermediate resolution (typically of the order of 50 nm, between light and electron microscopy) [213,214] in comparison with the high resolution of cryo-ET, has limited its use when fine ultrastructural details are needed. However, 15 nm spatial resolution has been already attained with this method [215].

This technique has provided further insights into the Vaccinia virus and Herpesvirus infection cycles [90,145] (Table 1).

#### 3.5.6. Cryo FIB-SEM

FIB-milling can be also used (as for resin-embedded cells, described above) for serial block imaging of (high-pressure)-frozen hydrated specimens in the SEM, generating large volume data for 3D analysis in an automated fashion [216] (Figure 4K). The lack of compression artifacts associated with cutting allows a more accurate merging of sequential images to get large datasets. Another advantage of this method, a common feature to all the approaches described above with the exception of CETOVIS, is its speed: imaging can start immediately after freezing. However, it is important to keep in mind that prolonged electron beam irradiation should be avoided to prevent modifications occurring on the sample surface.

Given the challenging aspects of these cryo-methods, to our knowledge only CET and X-ray cryo-tomography have been used so far to study virus-infected cells (Table 1).

## 4. Conclusions and Future Perspectives

3D-EM-based techniques follow the same principle: cells are “chopped” into sections of different thickness from which we obtain a number of 2D projections that are assembled into a 3D reconstruction of the biological object. Exceptions are cryo-ET and soft X-ray cryo-tomography, where entire cells are imaged, although limited to thin areas. In this regard, most of the methods described here are semi-destructive methods (e.g., ET, STEM tomography, serial sectioning), because the sections can be analyzed again at a later time point (e.g., at different magnifications or with another type of microscope) or even further used for immunocytochemical studies (serial sectioning), with the exception of SBEM or FIB-SEM, in which the slices are completely destroyed. However, the major advantage of the destructive sequential methods is that they do not suffer from warping, folding or loss of sections that can significantly affect the data quality and its subsequent analysis.

Foremost among the recent studies are those based on ET, which are increasingly used to elucidate the 3D architecture of virus-infected cells. FIB-SEM represents the most modern technique, although resolution in the Z-plane is still lower than with ET. However, the possibility to reconstruct complete cell volumes in an automatic manner provides unprecedented insights into the morphology of inter-cellular contacts as well as large cell organelles and their remodeling upon virus infection.

An important present/future direction is the use of multiple techniques to analyze the same sample. Thus, correlative light electron microscopy (CLEM) bridges the gap between light and electron microscopy. In this way information gained by fluorescence microscopy, such as colocalization of distinct proteins or rare events occurring in cells can be visualized by EM with high resolution, thus revealing the underlying subcellular structures. Furthermore, with EM we gain access to the so-called “space reference”, *i.e.*, not only the fluorescently labeled proteins are observed, but also their neighboring structures and even the whole cell. For all these reasons, CLEM is already an essential approach not only for virologists (e.g., [20,217]), but for cell biology in general (reviewed for example in [208,218,219,220,221]).

Along these lines, a recent multimodal approach named COIN (correlation optical and isotopic nanoscopy) combines three different imaging techniques: super-resolution microscopy, nano-SIMS (Secondary Ion Mass Spectrometry) [222] and EM, to provide information about the isotopic composition of many organelles and subcellular structures [223].

Furthermore, recent strides of cryo-EM (the so-called molecular microscopy) have shown that the molecular architecture of complexes can be resolved in their natural cellular environment, devoid of staining and chemical fixation artifacts. If multiple copies of these complexes are present the individual subvolumes containing them can be computationally averaged to improve the resolution to 2 nm (recently reviewed by [224,225]). Furthermore, in combination with advanced computational methods, such as molecular identification based on pattern recognition techniques [226,227], cryo-EM is currently the most promising approach to comprehensively map the macromolecular architecture of proteins or proteins assemblies (attained by X-ray crystallography or nuclear magnetic resonance -NMR- spectroscopy) inside the cryo-EM cellular tomograms (reviewed by [228]).

In spite of all these advancements, the main drawback of EM is its inability to study the dynamics of biological processes. As we have described in this review, cells must be fixed in order to be examined in the vacuum atmosphere of an electron microscope. However, some progress has been made towards observing the ultrastructure of living cells by means of *in situ* or environmental TEM. With this technique, cells can be examined in their native liquid environment (reviewed in [229,230]. For instance, by using this technique it has been possible to observe the assembly of rotavirus particles in a microfluidic platform [231]. The main advantage of this approach is that it circumvents the need for a dried, electron-conductive atmosphere. Although one of the inherent limitations of *in situ* TEM is that motion of the cells or viral particles in solution results in poor structural resolution [231], live cells engulfing nanoparticles have already been observed with this novel technique [232]. Therefore, we remain optimistic that EM-based methods providing time-resolved 3D information will become available in the not too distant future.

## References

[1] Zhou Z.H. (2008). Towards atomic resolution structural determination by single-particle cryo-electron microscopy. Curr. Opin. Struct. Biol..

[2] Cheng Y., Walz T. (2009). The advent of near-atomic resolution in single-particle electron microscopy. Annu. Rev. Biochem..

[3] Wolf M., Garcea R.L., Grigorieff N., Harrison S.C. (2010). Subunit interactions in bovine papillomavirus. Proc. Natl. Acad. Sci. USA.

[4] Bharat T.A., Davey N.E., Ulbrich P., Riches J.D., de Marco A., Rumlova M., Sachse C., Ruml T., Briggs J.A. (2012). Structure of the immature retroviral capsid at 8 A resolution by cryo-electron microscopy. Nature.

[5] Ruska E. (1987). Nobel lecture. The development of the electron microscope and of electron microscopy. Biosci. Rep..

[6] Biel S.S., Gelderblom H.R. (1999). Diagnostic electron microscopy is still a timely and rewarding method. J. Clin. Virol..

[7] Curry A., Appleton H., Dowsett B. (2006). Application of transmission electron microscopy to the clinical study of viral and bacterial infections: Present and future. Micron.

[8] Goldsmith C.S., Miller S.E. (2009). Modern uses of electron microscopy for detection of viruses. Clin. Microbiol. Rev..

[9] Gentile M., Gelderblom H.R. (2014). Electron microscopy in rapid viral diagnosis: An update. New Microbiol..

[10] Hazelton P.R., Gelderblom H.R. (2003). Electron microscopy for rapid diagnosis of infectious agents in emergent situations. Emerg. Infect. Dis..

[11] Roingeard P. (2008). Viral detection by electron microscopy: Past, present and future. Biol. Cell.

[12] Biel S.S., Madeley D. (2001). Diagnostic virology—The need for electron microscopy: A discussion paper. J. Clin. Virol..

[13] Risco C., de Castro I.F., Sanz-Sanchez L., Narayan K., Grandinetti G., Subramaniam S. (2014). Three-dimensional imaging of viral infections. Annu. Rev. Virol..

[14] Griffiths G. (1993). Fine Structure Immunocytochemistry.

[15] Hayat M.A. (1981). Fixation for Electron Microscopy.

[16] Steinbrecht R.A., Zierold K. (1987). Cryotechniques in Biological Electron Microscopy.

[17] Verkleij A.J., Leunissen J.L.M. (1989). Immuno-Gold Labeling in Cell Biology.

[18] Laue M. (2010). Electron microscopy of viruses. Methods Cell Biol..

[19] Flitney F.W. (1966). The time course of the fixation of albumin by formaldehyde, glutaraldehyde, acrolein and other higher aldehydes. J. R. Micros. Soc..

[20] Romero-Brey I., Merz A., Chiramel A., Lee J.Y., Chlanda P., Haselman U., Santarella-Mellwig R., Habermann A., Hoppe S., Kallis S. (2012). Three-dimensional architecture and biogenesis of membrane structures associated with hepatitis C virus replication. PLoS Pathog..

[21] Hayat M.A. (1981). The production of artifacts. Ultrastruct. Pathol..

[22] McDowall A.W., Chang J.J., Freeman R., Lepault J., Walter C.A., Dubochet J. (1983). Electron microscopy of frozen hydrated sections of vitreous ice and vitrified biological samples. J. Microsc..

[23] McDowall A., Gruenberg J., Romisch K., Griffiths G. (1989). The structure of organelles of the endocytic pathway in hydrated cryosections of cultured cells. Eur. J. Cell Biol..

[24] Small J.V. (1981). Organization of actin in the leading edge of cultured cells: Influence of osmium tetroxide and dehydration on the ultrastructure of actin meshworks. J. Cell Biol..

[25] Studer D., Humbel B.M., Chiquet M. (2008). Electron microscopy of high pressure frozen samples: Bridging the gap between cellular ultrastructure and atomic resolution. Histochem. Cell Biol..

[26] Adrian M., Dubochet J., Lepault J., McDowall A.W. (1984). Cryo-electron microscopy of viruses. Nature.

[27] Dubochet J., Adrian M., Chang J.J., Homo J.C., Lepault J., McDowall A.W., Schultz P. (1988). Cryo-electron microscopy of vitrified specimens. Q. Rev. Biophys..

[28] Muller M., Meister N., Moor H. (1980). Freezing in a propane jet and its application in freeze-fracturing. Mikroskopie.

[29] Giddings T.H., Staehelin L.A. (1980). Ribosome binding sites visualized on freeze-fractured membranes of the rough endoplasmic reticulum. J. Cell Biol..

[30] Espevik T., Elgsaeter A. (1981). In situ liquid propane jet-freezing and freeze-etching of monolayer cell cultures. J. Microsc..

[31] Swales L.S., Lane N.J. (1983). Insect intercellular junctions: Rapid freezing by jet propane. J. Cell Sci..

[32] Haggis G.H. (1986). Study of the conditions necessary for propane-jet freezing of fresh biological tissues without detectable ice formation. J. Microsc..

[33] Galway M.E., Heckman J.W., Hyde G.J., Fowke L.C. (1995). Advances in high-pressure and plunge-freeze fixation. Methods Cell Biol..

[34] Nitta K., Kaneko Y. (2004). Simple plunge freezing applied to plant tissues for capturing the ultrastructure close to the living state. J. Electron Microsc. (Tokyo).

[35] Richter T., Biel S.S., Sattler M., Wenck H., Wittern K.P., Wiesendanger R., Wepf R. (2007). Pros and cons: Cryo-electron microscopic evaluation of block faces *versus* cryo-sections from frozen-hydrated skin specimens prepared by different techniques. J. Microsc..

[36] Shimoni E., Muller M. (1998). On optimizing high-pressure freezing: From heat transfer theory to a new microbiopsy device. J. Microsc..

[37] Dubochet J. (2007). The physics of rapid cooling and its implications for cryoimmobilization of cells. Methods Cell Biol..

[38] Studer D., Michel M., Wohlwend M., Hunziker E.B., Buschmann M.D. (1995). Vitrification of articular cartilage by high-pressure freezing. J. Microsc..

[39] Allison D.P., Daw C.S., Rorvik M.C. (1987). The construction and operation of a simple inexpensive slam freezing device for electron microscopy. J. Microsc..

[40] Meryman H.T. (2007). Cryopreservation of living cells: Principles and practice. Transfusion.

[41] Moor H., Steinbrecht R.A., Zierold K. (1987). Theory and practice of high pressure freezing. Cryotechniques in Biological Electron Microscopy.

[42] Studer D., Michel M., Muller M. (1989). High pressure freezing comes of age. Scanning Microsc. Suppl..

[43] Vanhecke D., Graber W., Studer D. (2008). Close-to-native ultrastructural preservation by high pressure freezing. Methods Cell Biol..

[44] Al-Amoudi A., Dubochet J., Studer D. (2002). Amorphous solid water produced by cryosectioning of crystalline ice at 113 K. J. Microsc..

[45] Eppenberger-Eberhardt M., Riesinger I., Messerli M., Schwarb P., Muller M., Eppenberger H.M., Wallimann T. (1991). Adult rat cardiomyocytes cultured in creatine-deficient medium display large mitochondria with paracrystalline inclusions, enriched for creatine kinase. J. Cell Biol..

[46] Verkade P. (2008). Moving EM: The rapid transfer system as a new tool for correlative light and electron microscopy and high throughput for high-pressure freezing. J. Microsc..

[47] Kingsley R.E., Cole N.L. (1988). Preparation of cultured mammalian cells for transmission and scanning electron microscopy using Aclar film. J. Electron Microsc. Tech..

[48] Masurovsky E.B., Bunge R.P. (1989). Aclar film in biological electron mircoscopy. J. Electron Microsc. Tech..

[49] Jimenez N., Humbel B.M., van Donselaar E., Verkleij A.J., Burger K.N. (2006). Aclar discs: A versatile substrate for routine high-pressure freezing of mammalian cell monolayers. J. Microsc..

[50] Schauflinger M., Villinger C., Mertens T., Walther P., von Einem J. (2013). Analysis of human cytomegalovirus secondary envelopment by advanced electron microscopy. Cell Microbiol..

[51] Hohenberg H., Mannweiler K., Muller M. (1994). High-pressure freezing of cell suspensions in cellulose capillary tubes. J. Microsc..

[52] Sherman M.B., Trujillo J., Leahy I., Razmus D., Dehate R., Lorcheim P., Czarneski M.A., Zimmerman D., Newton J.T., Haddow A.D. (2013). Construction and organization of a BSL-3 cryo-electron microscopy laboratory at UTMB. J. Struct. Biol..

[53] Sosinsky G.E., Crum J., Jones Y.Z., Lanman J., Smarr B., Terada M., Martone M.E., Deerinck T.J., Johnson J.E., Ellisman M.H. (2008). The combination of chemical fixation procedures with high pressure freezing and freeze substitution preserves highly labile tissue ultrastructure for electron tomography applications. J. Struct. Biol..

[54] Fontana J., Lopez-Iglesias C., Tzeng W.P., Frey T.K., Fernandez J.J., Risco C. (2010). Three-dimensional structure of Rubella virus factories. Virology.

[55] Murk J.L., Posthuma G., Koster A.J., Geuze H.J., Verkleij A.J., Kleijmeer M.J., Humbel B.M. (2003). Influence of aldehyde fixation on the morphology of endosomes and lysosomes: Quantitative analysis and electron tomography. J. Microsc..

[56] Vale F.F., Correia A.C., Matos B., Moura Nunes J.F., Alves de Matos A.P., Mendez-Vilas A., Diaz J. (2010). Applications of transmission electron microscopy to virus detection and identification. Microscopy: Science. Technology, Applications and Education.

[57] Stoeckenius W., Mahr S.C. (1965). Studies on the reaction of osmium tetroxide with lipids and related compounds. Lab. Invest..

[58] Korn E.D. (1966). Structure of biological membranes. Science.

[59] Silva M.T., Guerra F.C., Magalhaes M.M. (1968). The fixative action of uranyl acetate in electron microscopy. Experientia.

[60] Nielson A.J., Griffith W.P. (1979). Tissue fixation by osmium tetroxide. A possible role for proteins. J. Histochem. Cytochem..

[61] Maupin-Szamier P., Pollard T.D. (1978). Actin filament destruction by osmium tetroxide. J. Cell Biol..

[62] Bendayan M., Zollinger M. (1983). Ultrastructural localization of antigenic sites on osmium-fixed tissues applying the protein A-gold technique. J. Histochem. Cytochem..

[63] FINCK H. (1960). Epoxy resins in electron microscopy. J. Biophys. Biochem. Cytol..

[64] Hawes P.C. (2015). Preparation of cultured cells using high-pressure freezing and freeze substitution for subsequent 2D or 3D visualization in the transmission electron microscope. Methods Mol. Biol..

[65] Hunziker E.B., Herrmann W., Schenk R.K., Mueller M., Moor H. (1984). Cartilage ultrastructure after high pressure freezing, freeze substitution, and low temperature embedding. I. Chondrocyte ultrastructure—Implications for the theories of mineralization and vascular invasion. J. Cell Biol..

[66] Humbel B., Müller M., O’Hare A.M.F., Müller M., Becker R.P., Boyde A., Wolosewick J.J. (1986). Freeze-substitution and low temperature embeding. The Science of Biological Specimen Preparation.

[67] Walther P., Ziegler A. (2002). Freeze substitution of high-pressure frozen samples: The visibility of biological membranes is improved when the substitution medium contains water. J. Microsc..

[68] Petsko G.A. (1975). Protein crystallography at sub-zero temperatures: Cryo-protective mother liquors for protein crystals. J. Mol. Biol..

[69] Carlemalm E., Armbruster B.L., Chiovetti R., Garavito R.M., Hobot J.A., Villiger W., Kellenberger E. (1982). Perspectives for achieving improved information by the observation of thin sections in the electron microscope. Tokai J. Exp. Clin. Med..

[70] Newman G.R., Jasani B., Williams E.D. (1983). A simple post-embedding system for the rapid demonstration of tissue antigens under the electron microscope. Histochem. J..

[71] Schwarz H., Humbel B.M. (1989). Influence of fixatives and embedding media on immunolabelling of freeze-substituted cells. Scanning Microsc. Suppl..

[72] Luby-Phelps K., Ning G., Fogerty J., Besharse J.C. (2003). Visualization of identified GFP-expressing cells by light and electron microscopy. J. Histochem. Cytochem..

[73] Nixon S.J., Webb R.I., Floetenmeyer M., Schieber N., Lo H.P., Parton R.G. (2009). A single method for cryofixation and correlative light, electron microscopy and tomography of zebrafish embryos. Traffic.

[74] McDonald K.L., Webb R.I. (2011). Freeze substitution in 3 hours or less. J. Microsc..

[75] Peddie C.J., Blight K., Wilson E., Melia C., Marrison J., Carzaniga R., Domart M.C., O’Toole P., Larijani B., Collinson L.M. (2014). Correlative and integrated light and electron microscopy of in-resin GFP fluorescence, used to localise diacylglycerol in mammalian cells. Ultramicroscopy.

[76] Kizilyaprak C., Longo G., Daraspe J., Humbel B.M. (2015). Investigation of resins suitable for the preparation of biological sample for 3-D electron microscopy. J. Struct. Biol..

[77] Reynolds E.S. (1963). The use of lead citrate at high pH as an electron-opaque stain in electron microscopy. J. Cell Biol..

[78] Briggman K.L., Bock D.D. (2012). Volume electron microscopy for neuronal circuit reconstruction. Curr. Opin. Neurobiol..

[79] Schwartz C.L., Sarbash V.I., Ataullakhanov F.I., McIntosh J.R., Nicastro D. (2007). Cryo-fluorescence microscopy facilitates correlations between light and cryo-electron microscopy and reduces the rate of photobleaching. J. Microsc..

[80] Fernandez-Moran H., Dahl A.O. (1952). Electron microscopy of ultrathin frozen sections of pollen grains. Science.

[81] Richter K. (1996). Aspects of cryofixation and cryosectioning for the observation of bulk biological samples in the hydrated state by cryoelectron microscopy. Scanning Microsc. Suppl..

[82] Frederik P.M., Busing W.M., Persson A. (1982). Concerning the nature of the cryosectioning process. J. Microsc..

[83] Frederik P.M., Busing W.M., Persson A. (1984). Surface defects on thin cryosections. Scan Electron Microsc..

[84] Frederik P.M., Bomans P.H., Stuart M.C. (1991). The ultrastructure of cryo-sections and intact vitrified cells—The effects of cryoprotectants and acceleration voltage on beam induced bubbling. Scanning Microsc. Suppl..

[85] Sitte H. (1996). Advanced instrumentation and methodology related to cryoultramicrotomy: A review. Scanning Microsc. Suppl..

[86] Al-Amoudi A., Chang J.J., Leforestier A., McDowall A., Salamin L.M., Norlen L.P., Richter K., Blanc N.S., Studer D., Dubochet J. (2004). Cryo-electron microscopy of vitreous sections. EMBO J..

[87] Chlanda P., Sachse M. (2014). Cryo-electron microscopy of vitreous sections. Methods Mol. Biol..

[88] Pierson J., Fernandez J.J., Bos E., Amini S., Gnaegi H., Vos M., Bel B., Adolfsen F., Carrascosa J.L., Peters P.J. (2010). Improving the technique of vitreous cryo-sectioning for cryo-electron tomography: Electrostatic charging for section attachment and implementation of an anti-contamination glove box. J. Struct. Biol..

[89] Elias H. (1972). Identification of structure by the common-sense approach. J. Microsc..

[90] Chichon F.J., Rodriguez M.J., Pereiro E., Chiappi M., Perdiguero B., Guttmann P., Werner S., Rehbein S., Schneider G., Esteban M. (2012). Cryo X-ray nano-tomography of vaccinia virus infected cells. J. Struct. Biol..

[91] Kopek B.G., Perkins G., Miller D.J., Ellisman M.H., Ahlquist P. (2007). Three-dimensional analysis of a viral RNA replication complex reveals a virus-induced mini-organelle. PLoS Biol..

[92] Baines J.D., Hsieh C.E., Wills E., Mannella C., Marko M. (2007). Electron tomography of nascent herpes simplex virus virions. J. Virol..

[93] Katayama S., Wei T., Omura T., Takagi J., Iwasaki K. (2007). Three-dimensional architecture of virus-packed tubule. J. Electron Microsc. (Tokyo).

[94] Sougrat R., Bartesaghi A., Lifson J.D., Bennett A.E., Bess J.W., Zabransky D.J., Subramaniam S. (2007). Electron tomography of the contact between T cells and SIV/HIV-1: Implications for viral entry. PLoS Pathog..

[95] Knoops K., Kikkert M., Worm S.H., Zevenhoven-Dobbe J.C., van der Meer Y., Koster A.J., Mommaas A.M., Snijder E.J. (2008). SARS-coronavirus replication is supported by a reticulovesicular network of modified endoplasmic reticulum. PLoS Biol..

[96] Carlson L.A., Briggs J.A., Glass B., Riches J.D., Simon M.N., Johnson M.C., Muller B., Grunewald K., Krausslich H.G. (2008). Three-dimensional analysis of budding sites and released virus suggests a revised model for HIV-1 morphogenesis. Cell Host. Microb..

[97] Majorovits E., Nejmeddine M., Tanaka Y., Taylor G.P., Fuller S.D., Bangham C.R. (2008). Human T-lymphotropic virus-1 visualized at the virological synapse by electron tomography. PLoS ONE.

[98] Chichon F.J., Rodriguez M.J., Risco C., Fraile-Ramos A., Fernandez J.J., Esteban M., Carrascosa J.L. (2009). Membrane remodelling during vaccinia virus morphogenesis. Biol. Cell.

[99] Welsch S., Miller S., Romero-Brey I., Merz A., Bleck C.K., Walther P., Fuller S.D., Antony C., Krijnse-Locker J., Bartenschlager R. (2009). Composition and three-dimensional architecture of the dengue virus replication and assembly sites. Cell Host. Microb..

[100] Chlanda P., Carbajal M.A., Cyrklaff M., Griffiths G., Krijnse-Locker J. (2009). Membrane rupture generates single open membrane sheets during vaccinia virus assembly. Cell Host. Microb..

[101] Wei T., Uehara-Ichiki T., Miyazaki N., Hibino H., Iwasaki K., Omura T. (2009). Association of Rice gall dwarf virus with microtubules is necessary for viral release from cultured insect vector cells. J. Virol..

[102] Peng L., Ryazantsev S., Sun R., Zhou Z.H. (2010). Three-dimensional visualization of gammaherpesvirus life cycle in host cells by electron tomography. Structure.

[103] Gillespie L.K., Hoenen A., Morgan G., Mackenzie J.M. (2010). The endoplasmic reticulum provides the membrane platform for biogenesis of the flavivirus replication complex. J. Virol..

[104] Gangodkar S., Jain P., Dixit N., Ghosh K., Basu A. (2010). Dengue virus-induced autophagosomes and changes in endomembrane ultrastructure imaged by electron tomography and whole-mount grid-cell culture techniques. J. Electron Microsc. (Tokyo).

[105] Knoops K., Swett-Tapia C., van den Worm S.H., Te Velthuis A.J., Koster A.J., Mommaas A.M., Snijder E.J., Kikkert M. (2010). Integrity of the early secretory pathway promotes, but is not required for, severe acute respiratory syndrome coronavirus RNA synthesis and virus-induced remodeling of endoplasmic reticulum membranes. J. Virol..

[106] Welsch S., Kolesnikova L., Krahling V., Riches J.D., Becker S., Briggs J.A. (2010). Electron tomography reveals the steps in filovirus budding. PLoS Pathog..

[107] Soonsawad P., Xing L., Milla E., Espinoza J.M., Kawano M., Marko M., Hsieh C., Furukawa H., Kawasaki M., Weerachatyanukul W. (2010). Structural evidence of glycoprotein assembly in cellular membrane compartments prior to Alphavirus budding. J. Virol..

[108] Limpens R.W., van der Schaar H.M., Kumar D., Koster A.J., Snijder E.J., van Kuppeveld F.J., Barcena M. (2011). The transformation of enterovirus replication structures: A three-dimensional study of single- and double-membrane compartments. MBio.

[109] Wei T., Miyazaki N., Uehara-Ichiki T., Hibino H., Shimizu T., Netsu O., Kikuchi A., Sasaya T., Iwasaki K., Omura T. (2011). Three-dimensional analysis of the association of viral particles with mitochondria during the replication of Rice gall dwarf virus. J. Mol. Biol..

[110] Knoops K., Barcena M., Limpens R.W., Koster A.J., Mommaas A.M., Snijder E.J. (2012). Ultrastructural characterization of arterivirus replication structures: Reshaping the endoplasmic reticulum to accommodate viral RNA synthesis. J. Virol..

[111] Belov G.A., Nair V., Hansen B.T., Hoyt F.H., Fischer E.R., Ehrenfeld E. (2012). Complex dynamic development of poliovirus membranous replication complexes. J. Virol..

[112] Offerdahl D.K., Dorward D.W., Hansen B.T., Bloom M.E. (2012). A three-dimensional comparison of tick-borne flavivirus infection in mammalian and tick cell lines. PLoS ONE.

[113] Miorin L., Romero-Brey I., Maiuri P., Hoppe S., Krijnse-Locker J., Bartenschlager R., Marcello A. (2013). Three-dimensional architecture of tick-borne encephalitis virus replication sites and trafficking of the replicated RNA. J. Virol..

[114] Maier H.J., Hawes P.C., Cottam E.M., Mantell J., Verkade P., Monaghan P., Wileman T., Britton P. (2013). Infectious bronchitis virus generates spherules from zippered endoplasmic reticulum membranes. MBio.

[115] Suarez C., Welsch S., Chlanda P., Hagen W., Hoppe S., Kolovou A., Pagnier I., Raoult D., Krijnse L.J. (2013). Open membranes are the precursors for assembly of large DNA viruses. Cell Microbiol..

[116] Hoenen A., Gillespie L., Morgan G., van der Heide P., Khromykh A., Mackenzie J. (2014). The West Nile virus assembly process evades the conserved antiviral mechanism of the interferon-induced MXA protein. Virology.

[117] Junjhon J., Pennington J.G., Edwards T.J., Perera R., Lanman J., Kuhn R.J. (2014). Ultrastructural characterization and three-dimensional architecture of replication sites in dengue virus-infected mosquito cells. J. Virol..

[118] Cao X., Jin X., Zhang X., Li Y., Wang C., Wang X., Hong J., Wang X., Li D., Zhang Y. (2015). Morphogenesis of Endoplasmic Reticulum Membrane-Invaginated Vesicles during Beet Black Scorch Virus Infection: Role of Auxiliary Replication Protein and New Implications of Three-Dimensional Architecture. J. Virol..

[119] Ladinsky M.S., Kieffer C., Olson G., Deruaz M., Vrbanac V., Tager A.M., Kwon D.S., Bjorkman P.J. (2014). Electron tomography of HIV-1 infection in gut-associated lymphoid tissue. PLoS Pathog..

[120] Whiteman M.C., Popov V., Sherman M.B., Wen J., Barrett A.D. (2015). Attenuated West Nile virus mutant NS1130-132QQA/175A/207A exhibits virus-induced ultrastructural changes and accumulation of protein in the endoplasmic reticulum. J. Virol..

[121] Shi Y., Li K., Tang P., Li Y., Zhou Q., Yang K., Zhang Q. (2015). Three-dimensional visualization of the Autographa californica multiple nucleopolyhedrovirus occlusion-derived virion envelopment process gives new clues as to its mechanism. Virology.

[122] Bily T., Palus M., Eyer L., Elsterova J., Vancova M., Ruzek D. (2015). Electron Tomography Analysis of Tick-Borne Encephalitis Virus Infection in Human Neurons. Sci. Rep..

[123] Suarez C., Andres G., Kolovou A., Hoppe S., Salas M.L., Walther P., Krijnse L.J. (2015). African swine fever virus assembles a single membrane derived from rupture of the endoplasmic reticulum. Cell Microbiol..

[124] Mutsafi Y., Shimoni E., Shimon A., Minsky A. (2013). Membrane assembly during the infection cycle of the giant Mimivirus. PLoS Pathog..

[125] Milrot E., Mutsafi Y., Fridmann-Sirkis Y., Shimoni E., Rechav K., Gurnon J.R., van Etten J.L., Minsky A. (2015). Virus-host interactions: Insights from the replication cycle of the large Paramecium bursaria chlorella virus. Cell Microbiol..

[126] Fontana J., Lopez-Montero N., Elliott R.M., Fernandez J.J., Risco C. (2008). The unique architecture of Bunyamwera virus factories around the Golgi complex. Cell Microbiol..

[127] Reichelt M., Joubert L., Perrino J., Koh A.L., Phanwar I., Arvin A.M. (2012). 3D reconstruction of VZV infected cell nuclei and PML nuclear cages by serial section array scanning electron microscopy and electron tomography. PLoS Pathog..

[128] Ferraris P., Beaumont E., Uzbekov R., Brand D., Gaillard J., Blanchard E., Roingeard P. (2013). Sequential biogenesis of host cell membrane rearrangements induced by hepatitis C virus infection. Cell Mol. Life Sci..

[129] Fernandez d.C.I., Zamora P.F., Ooms L., Fernandez J.J., Lai C.M., Mainou B.A., Dermody T.S., Risco C. (2014). Reovirus forms neo-organelles for progeny particle assembly within reorganized cell membranes. MBio.

[130] Zellnig G., Pockl M.H., Mostl S., Zechmann B. (2014). Two and three dimensional characterization of Zucchini Yellow Mosaic Virus induced structural alterations in Cucurbita pepo L. plants. J. Struct. Biol..

[131] Bennett A.E., Narayan K., Shi D., Hartnell L.M., Gousset K., He H., Lowekamp B.C., Yoo T.S., Bliss D., Freed E.O., Subramaniam S. (2009). Ion-abrasion scanning electron microscopy reveals surface-connected tubular conduits in HIV-infected macrophages. PLoS Pathog..

[132] Felts R.L., Narayan K., Estes J.D., Shi D., Trubey C.M., Fu J., Hartnell L.M., Ruthel G.T., Schneider D.K., Nagashima K. (2010). 3D visualization of HIV transfer at the virological synapse between dendritic cells and T cells. Proc. Natl. Acad. Sci. USA.

[133] Do T., Murphy G., Earl L.A., del Prete G.Q., Grandinetti G., Li G.H., Estes J.D., Rao P., Trubey C.M., Thomas J. (2014). Three-dimensional imaging of HIV-1 virological synapses reveals membrane architectures involved in virus transmission. J. Virol..

[134] Cyrklaff M., Linaroudis A., Boicu M., Chlanda P., Baumeister W., Griffiths G., Krijnse-Locker J. (2007). Whole cell cryo-electron tomography reveals distinct disassembly intermediates of vaccinia virus. PLoS ONE.

[135] Maurer U.E., Sodeik B., Grunewald K. (2008). Native 3D intermediates of membrane fusion in herpes simplex virus 1 entry. Proc. Natl. Acad. Sci. USA.

[136] Carlson L.A., de Marco A., Oberwinkler H., Habermann A., Briggs J.A., Krausslich H.G., Grunewald K. (2010). Cryo electron tomography of native HIV-1 budding sites. PLoS Pathog..

[137] Bharat T.A., Riches J.D., Kolesnikova L., Welsch S., Krahling V., Davey N., Parsy M.L., Becker S., Briggs J.A. (2011). Cryo-electron tomography of Marburg virus particles and their morphogenesis within infected cells. PLoS Biol..

[138] Ibiricu I., Huiskonen J.T., Dohner K., Bradke F., Sodeik B., Grunewald K. (2011). Cryo electron tomography of herpes simplex virus during axonal transport and secondary envelopment in primary neurons. PLoS Pathog..

[139] Vijayakrishnan S., Loney C., Jackson D., Suphamungmee W., Rixon F.J., Bhella D. (2013). Cryotomography of budding influenza A virus reveals filaments with diverse morphologies that mostly do not bear a genome at their distal end. PLoS Pathog..

[140] Hu B., Margolin W., Molineux I.J., Liu J. (2013). The bacteriophage t7 virion undergoes extensive structural remodeling during infection. Science.

[141] Miyazaki N., Akita F., Nakagawa A., Murata K., Omura T., Iwasaki K. (2013). Cryo-electron tomography: Moving towards revealing the viral life cycle of Rice dwarf virus. J. Synchrotron. Radiat..

[142] Dai W., Fu C., Raytcheva D., Flanagan J., Khant H.A., Liu X., Rochat R.H., Haase-Pettingell C., Piret J., Ludtke S.J. (2013). Visualizing virus assembly intermediates inside marine cyanobacteria. Nature.

[143] Mueller J., Pfanzelter J., Winkler C., Narita A., Le C.C., Nemethova M., Carlier M.F., Maeda Y., Welch M.D., Ohkawa T. (2014). Electron tomography and simulation of baculovirus actin comet tails support a tethered filament model of pathogen propulsion. PLoS Biol..

[144] Woodward C.L., Cheng S.N., Jensen G.J. (2015). Electron cryotomography studies of maturing HIV-1 particles reveal the assembly pathway of the viral core. J. Virol..

[145] Hagen C., Guttmann P., Klupp B., Werner S., Rehbein S., Mettenleiter T.C., Schneider G., Grunewald K. (2012). Correlative VIS-fluorescence and soft X-ray cryo-microscopy/tomography of adherent cells. J. Struct. Biol..

[146] Koster A.J., Grimm R., Typke D., Hegerl R., Stoschek A., Walz J., Baumeister W. (1997). Perspectives of molecular and cellular electron tomography. J. Struct. Biol..

[147] Baumeister W., Grimm R., Walz J. (1999). Electron tomography of molecules and cells. Trends Cell Biol..

[148] Lucic V., Forster F., Baumeister W. (2005). Structural studies by electron tomography: From cells to molecules. Annu. Rev. Biochem..

[149] De Rosier D.J., Klug A. (1968). Reconstruction of three dimensional structures from electron micrographs. Nature.

[150] Hart R.G. (1968). Electron microscopy of unstained biological material: The polytropic montage. Science.

[151] Radon J. (1917). Über die Bestimmung von Funktionen durch ihre Integralwerte längs gewisser Mannigfaltigkeiten. Sächs. Akad. Wiss. Leipzig Math. Phys. Kl..

[152] McDonald K.L., Auer M. (2006). High-pressure freezing, cellular tomography, and structural cell biology. Biotechniques.

[153] Penczek P., Marko M., Buttle K., Frank J. (1995). Double-tilt electron tomography. Ultramicroscopy.

[154] Mastronarde D.N. (1997). Dual-axis tomography: An approach with alignment methods that preserve resolution. J. Struct. Biol..

[155] Hoog J.L., Schwartz C., Noon A.T., O'Toole E.T., Mastronarde D.N., McIntosh J.R., Antony C. (2007). Organization of interphase microtubules in fission yeast analyzed by electron tomography. Dev. Cell.

[156] Noske A.B., Costin A.J., Morgan G.P., Marsh B.J. (2008). Expedited approaches to whole cell electron tomography and organelle mark-up in situ in high-pressure frozen pancreatic islets. J. Struct. Biol..

[157] Luther P.K., Lawrence M.C., Crowther R.A. (1988). A method for monitoring the collapse of plastic sections as a function of electron dose. Ultramicroscopy.

[158] Iwasaki K., Omura T. (2010). Electron tomography of the supramolecular structure of virus-infected cells. Curr. Opin. Struct. Biol..

[159] Romero-Brey I., Bartenschlager R. (2014). Membranous replication factories induced by plus-strand RNA viruses. Viruses.

[160] Biskupek J., Leschner J., Walther P., Kaiser U. (2010). Optimization of STEM tomography acquisition—A comparison of convergent beam and parallel beam STEM tomography. Ultramicroscopy.

[161] Kellenberger E., Carlemalm E., Villiger W., Wurtz M., Mory C., Colliex C. (1986). Z-contrast in biology. A comparison with other imaging modes. Ann. N. Y. Acad. Sci..

[162] Yakushevska A.E., Lebbink M.N., Geerts W.J., Spek L., van Donselaar E.G., Jansen K.A., Humbel B.M., Post J.A., Verkleij A.J., Koster A.J. (2007). STEM tomography in cell biology. J. Struct. Biol..

[163] Aoyama K., Takagi T., Hirase A., Miyazawa A. (2008). STEM tomography for thick biological specimens. Ultramicroscopy.

[164] Hohmann-Marriott M.F., Sousa A.A., Azari A.A., Glushakova S., Zhang G., Zimmerberg J., Leapman R.D. (2009). Nanoscale 3D cellular imaging by axial scanning transmission electron tomography. Nat. Methods.

[165] Hohn K., Sailer M., Wang L., Lorenz M., Schneider M.E., Walther P. (2011). Preparation of cryofixed cells for improved 3D ultrastructure with scanning transmission electron tomography. Histochem. Cell Biol..

[166] Sousa A.A., Leapman R.D. (2012). Development and application of STEM for the biological sciences. Ultramicroscopy.

[167] SJOSTRAND F.S. (1958). Ultrastructure of retinal rod synapses of the guinea pig eye as revealed by three-dimensional reconstructions from serial sections. J. Ultrastruct. Res..

[168] White J.G., Southgate E., Thomson J.N., Brenner S. (1986). The structure of the nervous system of the nematode Caenorhabditis elegans. Philos. Trans. R. Soc. Lond B Biol. Sci..

[169] Bumbarger D.J., Riebesell M., Rodelsperger C., Sommer R.J. (2013). System-wide rewiring underlies behavioral differences in predatory and bacterial-feeding nematodes. Cell.

[170] Micheva K.D., Smith S.J. (2007). Array tomography: A new tool for imaging the molecular architecture and ultrastructure of neural circuits. Neuron.

[171] Saalfeld S., Cardona A., Hartenstein V., Tomancak P. (2010). As-rigid-as-possible mosaicking and serial section registration of large ssTEM datasets. Bioinformatics..

[172] Kaynig V., Fischer B., Muller E., Buhmann J.M. (2010). Fully automatic stitching and distortion correction of transmission electron microscope images. J. Struct. Biol..

[173] Hayworth K.J., Kasthuri N., Schalek R., Lichtman J.W. (2006). Automating the Collection of Ultrathin Serial Sections for Large Volume TEM Reconstructions. Microsc. Microanal..

[174] Hayworth K.J., Morgan J.L., Schalek R., Berger D.R., Hildebrand D.G., Lichtman J.W. (2014). Imaging ATUM ultrathin section libraries with WaferMapper: A multi-scale approach to EM reconstruction of neural circuits. Front. Neural. Circuits.

[175] Schauflinger M., Villinger C., Walther P. (2013). Three-dimensional visualization of virus-infected cells by serial sectioning: An electron microscopic study using resin embedded cells. Methods Mol. Biol..

[176] Risco C., Fernandez D.C.I. (2013). Virus morphogenesis in the cell: Methods and observations. Subcell. Biochem..

[177] Denk W., Horstmann H. (2004). Serial block-face scanning electron microscopy to reconstruct three-dimensional tissue nanostructure. PLoS Biol..

[178] Heymann J.A., Hayles M., Gestmann I., Giannuzzi L.A., Lich B., Subramaniam S. (2006). Site-specific 3D imaging of cells and tissues with a dual beam microscope. J. Struct. Biol..

[179] Hekking L.H., Lebbink M.N., de Winter D.A., Schneijdenberg C.T., Brand C.M., Humbel B.M., Verkleij A.J., Post J.A. (2009). Focused ion beam-scanning electron microscope: Exploring large volumes of atherosclerotic tissue. J. Microsc..

[180] Villinger C., Schauflinger M., Gregorius H., Kranz C., Hohn K., Nafeey S., Walther P. (2014). Three-dimensional imaging of adherent cells using FIB/SEM and STEM. Methods Mol. Biol..

[181] Narayan K., Danielson C.M., Lagarec K., Lowekamp B.C., Coffman P., Laquerre A., Phaneuf M.W., Hope T.J., Subramaniam S. (2014). Multi-resolution correlative focused ion beam scanning electron microscopy: Applications to cell biology. J. Struct. Biol..

[182] Knott G., Rosset S., Cantoni M. (2011). Focussed ion beam milling and scanning electron microscopy of brain tissue. J. Vis. Exp..

[183] Villinger C., Gregorius H., Kranz C., Hohn K., Munzberg C., von W.G., Mizaikoff B., Wanner G., Walther P. (2012). FIB/SEM tomography with TEM-like resolution for 3D imaging of high-pressure frozen cells. Histochem. Cell Biol..

[184] Medalia O., Weber I., Frangakis A.S., Nicastro D., Gerisch G., Baumeister W. (2002). Macromolecular architecture in eukaryotic cells visualized by cryoelectron tomography. Science.

[185] Baumeister W. (2004). Mapping molecular landscapes inside cells. Biol. Chem..

[186] Hsieh C.E., Marko M., Frank J., Mannella C.A. (2002). Electron tomographic analysis of frozen-hydrated tissue sections. J. Struct. Biol..

[187] Frank J., Wagenknecht T., McEwen B.F., Marko M., Hsieh C.E., Mannella C.A. (2002). Three-dimensional imaging of biological complexity. J. Struct. Biol..

[188] Zhang P., Weis R.M., Peters P.J., Subramaniam S. (2007). Electron tomography of bacterial chemotaxis receptor assemblies. Methods Cell Biol..

[189] Al-Amoudi A., Diez D.C., Betts M.J., Frangakis A.S. (2007). The molecular architecture of cadherins in native epidermal desmosomes. Nature.

[190] Norlen L., Oktem O., Skoglund U. (2009). Molecular cryo-electron tomography of vitreous tissue sections: Current challenges. J. Microsc..

[191] Zuber B., Nikonenko I., Klauser P., Muller D., Dubochet J. (2005). The mammalian central nervous synaptic cleft contains a high density of periodically organized complexes. Proc. Natl. Acad. Sci. USA.

[192] Gruska M., Medalia O., Baumeister W., Leis A. (2008). Electron tomography of vitreous sections from cultured mammalian cells. J. Struct. Biol..

[193] Al-Amoudi A., Castano-Diez D., Devos D.P., Russell R.B., Johnson G.T., Frangakis A.S. (2011). The three-dimensional molecular structure of the desmosomal plaque. Proc. Natl. Acad. Sci. USA.

[194] Al-Amoudi A., Studer D., Dubochet J. (2005). Cutting artefacts and cutting process in vitreous sections for cryo-electron microscopy. J. Struct. Biol..

[195] Dubochet J. (2012). Cryo-EM—The first thirty years. J. Microsc..

[196] Marko M., Hsieh C., Schalek R., Frank J., Mannella C. (2007). Focused-ion-beam thinning of frozen-hydrated biological specimens for cryo-electron microscopy. Nat. Methods.

[197] Rigort A., Bauerlein F.J., Villa E., Eibauer M., Laugks T., Baumeister W., Plitzko J.M. (2012). Focused ion beam micromachining of eukaryotic cells for cryoelectron tomography. Proc. Natl. Acad. Sci. USA.

[198] Hsieh C., Schmelzer T., Kishchenko G., Wagenknecht T., Marko M. (2014). Practical workflow for cryo focused-ion-beam milling of tissues and cells for cryo-TEM tomography. J. Struct. Biol..

[199] Mahamid J., Schampers R., Persoon H., Hyman A.A., Baumeister W., Plitzko J.M. (2015). A focused ion beam milling and lift-out approach for site-specific preparation of frozen-hydrated lamellas from multicellular organisms. J. Struct. Biol..

[200] Koning R.I., Koster A.J. (2013). Cellular nanoimaging by cryo electron tomography. Methods Mol. Biol..

[201] Grunewald K., Cyrklaff M. (2006). Structure of complex viruses and virus-infected cells by electron cryo tomography. Curr. Opin. Microbiol..

[202] Zeev-Ben-Mordehai T., Hagen C., Grunewald K. (2014). A cool hybrid approach to the herpesvirus “life” cycle. Curr. Opin. Virol..

[203] Jones A.V., Leonard K.R. (1978). Scanning transmission electron microscopy of unstained biological sections. Nature.

[204] Takaoka A., Hasegawa T. (2006). Observations of unstained biological specimens using a low-energy, high-resolution STEM. J. Electron Microsc. (Tokyo).

[205] Buban J.P., Ramasse Q., Gipson B., Browning N.D., Stahlberg H. (2010). High-resolution low-dose scanning transmission electron microscopy. J. Electron Microsc. (Tokyo).

[206] Wolf S.G., Houben L., Elbaum M. (2014). Cryo-scanning transmission electron tomography of vitrified cells. Nat. Methods.

[207] McDermott G., Fox D.M., Epperly L., Wetzler M., Barron A.E., Le Gros M.A., Larabell C.A. (2012). Visualizing and quantifying cell phenotype using soft X-ray tomography. Bioessays.

[208] Schneider G., Guttmann P., Rehbein S., Werner S., Follath R. (2012). Cryo X-ray microscope with flat sample geometry for correlative fluorescence and nanoscale tomographic imaging. J. Struct. Biol..

[209] Kirz J., Jacobsen C., Howells M. (1995). Soft X-ray microscopes and their biological applications. Q. Rev. Biophys..

[210] Schmahl G., Rudolph D., Niemann B., Guttmann P., Thieme J., Schneider G. (1996). X-ray microscopy. Naturwissenschaften.

[211] Meyer-Ilse W., Hamamoto D., Nair A., Lelievre S.A., Denbeaux G., Johnson L., Pearson A.L., Yager D., Legros M.A., Larabell C.A. (2001). High resolution protein localization using soft X-ray microscopy. J. Microsc..

[212] Chapman H.N., Jacobsen C., Williams S. (1996). A characterisation of dark-field imaging of colloidal gold labels in a scanning transmission X-ray microscope. Ultramicroscopy.

[213] Weiss D., Schneider G., Niemann B., Guttmann P., Rudolph D., Schmahl G. (2000). Computed tomography of cryogenic biological specimens based on X-ray microscopic images. Ultramicroscopy.

[214] Gu W., Etkin L.D., Le Gros M.A., Larabell C.A. (2007). X-ray tomography of Schizosaccharomyces pombe. Differentiation.

[215] Chao W., Harteneck B.D., Liddle J.A., Anderson E.H., Attwood D.T. (2005). Soft X-ray microscopy at a spatial resolution better than 15 nm. Nature.

[216] Schertel A., Snaidero N., Han H.M., Ruhwedel T., Laue M., Grabenbauer M., Mobius W. (2013). Cryo FIB-SEM: Volume imaging of cellular ultrastructure in native frozen specimens. J. Struct. Biol..

[217] Jun S., Ke D., Debiec K., Zhao G., Meng X., Ambrose Z., Gibson G.A., Watkins S.C., Zhang P. (2011). Direct visualization of HIV-1 with correlative live-cell microscopy and cryo-electron tomography. Structure..

[218] Sartori A., Gatz R., Beck F., Rigort A., Baumeister W., Plitzko J.M. (2007). Correlative microscopy: Bridging the gap between fluorescence light microscopy and cryo-electron tomography. J. Struct. Biol..

[219] Lucas M.S., Gunthert M., Gasser P., Lucas F., Wepf R. (2012). Bridging microscopes: 3D correlative light and scanning electron microscopy of complex biological structures. Methods Cell Biol..

[220] De Boer P., Hoogenboom J.P., Giepmans B.N. (2015). Correlated light and electron microscopy: Ultrastructure lights up!. Nat. Methods.

[221] Loussert F.C., Humbel B.M. (2015). Correlative microscopy. Arch. Biochem. Biophys..

[222] Steinhauser M.L., Bailey A.P., Senyo S.E., Guillermier C., Perlstein T.S., Gould A.P., Lee R.T., Lechene C.P. (2012). Multi-isotope imaging mass spectrometry quantifies stem cell division and metabolism. Nature.

[223] Saka S.K., Vogts A., Krohnert K., Hillion F., Rizzoli S.O., Wessels J.T. (2014). Correlated optical and isotopic nanoscopy. Nat. Commun..

[224] Briggs J.A. (2013). Structural biology in situ--the potential of subtomogram averaging. Curr. Opin. Struct. Biol..

[225] Hoenger A. (2014). High-resolution cryo-electron microscopy on macromolecular complexes and cell organelles. Protoplasma.

[226] Bohm J., Frangakis A.S., Hegerl R., Nickell S., Typke D., Baumeister W. (2000). Toward detecting and identifying macromolecules in a cellular context: Template matching applied to electron tomograms. Proc. Natl. Acad. Sci. USA.

[227] Frangakis A.S., Bohm J., Forster F., Nickell S., Nicastro D., Typke D., Hegerl R., Baumeister W. (2002). Identification of macromolecular complexes in cryoelectron tomograms of phantom cells. Proc. Natl. Acad. Sci. USA.

[228] Milne J.L., Borgnia M.J., Bartesaghi A., Tran E.E., Earl L.A., Schauder D.M., Lengyel J., Pierson J., Patwardhan A., Subramaniam S. (2013). Cryo-electron microscopy--a primer for the non-microscopist. FEBS J..

[229] De Jonqe N., Ross F.M. (2011). Electron microscopy of specimens in liquid. Nat. Nanotechnol..

[230] Dukes M.J., Gilmore B.L., Tanner J.R., McDonald S.M., Kelly D.F. (2013). In situ TEM of biological assemblies in liquid. J. Vis. Exp..

[231] Gilmore B.L., Showalter S.P., Dukes M.J., Tanner J.R., Demmert A.C., McDonald S.M., Kelly D.F. (2013). Visualizing viral assemblies in a nanoscale biosphere. Lab Chip..

[232] Peckys D.B., de Jonge N. (2011). Visualizing gold nanoparticle uptake in live cells with liquid scanning transmission electron microscopy. Nano. Lett..

